# Tenascin C^+^ papillary fibroblasts facilitate neuro-immune interaction in a mouse model of psoriasis

**DOI:** 10.1038/s41467-023-37798-x

**Published:** 2023-04-10

**Authors:** Xiaojie Cai, Maoying Han, Fangzhou Lou, Yang Sun, Qianqian Yin, Libo Sun, Zhikai Wang, Xiangxiao Li, Hong Zhou, Zhenyao Xu, Hong Wang, Siyu Deng, Xichen Zheng, Taiyu Zhang, Qun Li, Bin Zhou, Honglin Wang

**Affiliations:** 1 Precision Research Center for Refractory Diseases, Shanghai General Hospital, Shanghai Jiao Tong University School of Medicine, Shanghai, 201620 China; 2 State Key Laboratory of Cell Biology, CAS Center for Excellence in Molecular Cell Science, Shanghai Institute of Biochemistry and Cell Biology, Chinese Academy of Sciences, University of Chinese Academy of Sciences, Shanghai, 200031 China; 3 Shanghai Institute of Immunology, Shanghai Jiao Tong University School of Medicine, Shanghai, 200025 China; 4 The Department of Cardiovascular Medicine, State Key Laboratory of Medical Genomics, Ruijin Hospital, Shanghai Institute of Hypertension, Shanghai Jiao Tong University School of Medicine, Shanghai, 200025 China

**Keywords:** Chronic inflammation, Psoriasis

## Abstract

Dermal fibroblasts and cutaneous nerves are important players in skin diseases, while their reciprocal roles during skin inflammation have not been characterized. Here we identify an inflammation-induced subset of papillary fibroblasts that promotes aberrant neurite outgrowth and psoriasiform skin inflammation by secreting the extracellular matrix protein tenascin-C (TNC). Single-cell analysis of fibroblast lineages reveals a *Tnc*^+^ papillary fibroblast subset with pro-axonogenesis and neuro-regulation transcriptomic hallmarks. TNC overexpression in fibroblasts boosts neurite outgrowth in co-cultured neurons, while fibroblast-specific TNC ablation suppresses hyperinnervation and alleviates skin inflammation in male mice modeling psoriasis. Dermal γδT cells, the main producers of type 17 pathogenic cytokines, frequently contact nerve fibers in mouse psoriasiform lesions and are likely modulated by postsynaptic signals. Overall, our results highlight the role of an inflammation-responsive fibroblast subset in facilitating neuro-immune synapse formation and suggest potential avenues for future therapeutic research.

## Abstract (web summary)

Local cues for hyperinnervation in chronic skin diseases are not fully understood. Here, the authors show that a distinct subset of dermal papillary fibroblasts promote neurite outgrowth and facilitate neuron-immune interactions through extracellular matrix remodeling in a mouse model of psoriasis

## Introduction

Dermal fibroblasts are heterogeneous in their anatomical locations and functions in skin homeostasis and diseases^1–3^. Histologically, papillary fibroblasts beneath the epidermis can be easily distinguished from reticular fibroblasts in the lower dermis, and there are special fibroblast subpopulations that support skin appendages such as dermal papillae^4^. In addition to anatomical distinctions, dermal fibroblasts can be further identified into functional subsets according to their biological features during skin inflammation, immunity, repair, and aging^5–9^. Mounting evidence indicates that distinct fibroblast subsets participate in skin pathophysiology as local pro-inflammatory cues through the regulation of immune cells via cytokine/chemokine secretion^6,7^ and the production of extracellular matrix proteins^9,10^. Identification of these pathogenic fibroblast subsets provides novel therapeutic avenues for multiple skin diseases, including type 2 dermatitis^6^, autoimmunity^7^, and fibrosis^8^. However, the heterogeneity and functions of fibroblasts in psoriasis remain unexplored.

Psoriasis is a chronic, immune-mediated skin disease with a global prevalence of 2–3%^11^. The most common manifestation, psoriasis vulgaris, is characterized by sharply demarcated, scaly, erythematous plaques in the skin^12^. The interleukin (IL)−23/IL-17 axis is pivotal in psoriasis pathogenesis^13^ and makes psoriasis a type 17 skin inflammation. Recently, cutaneous sensory nerves have been reported to play crucial roles in driving IL-23 production and type 17 T (T17) cell activation^14^. Hyperinnervation is a common feature of psoriatic lesions^15–17^, while denervation or peripheral nerve blockade ameliorates psoriasis in humans and rodents^18,19^. Optogenetic activation of TRPV1^+^ sensory nerves is sufficient to induce a type 17 inflammatory response in skin^20^. Besides, cutaneous nerves interact with multiple immune cells^21,22^, contributing to the severity and manifestation of skin lesions. With plentiful supportive studies of neuro-immune communication, little is known about the local cues that promote innervation during skin inflammation.

In this work, we take psoriasis as a model of type 17 skin inflammation and investigate the heterogeneity and potential functions of dermal fibroblast lineages in this disease which are previously uncharacterized. Aside from global skews in histology and transcriptional features, we identify an inflammation-induced papillary fibroblast subset that promotes neurite outgrowth, facilitates neuro-immune crosstalk and exacerbates psoriasiform lesions, providing further insights into stroma–neuron interactions underlying skin inflammation.

## Results

### Dermal fibroblasts show altered quantity and distribution adapt to inflammation

**Fig. 1:**
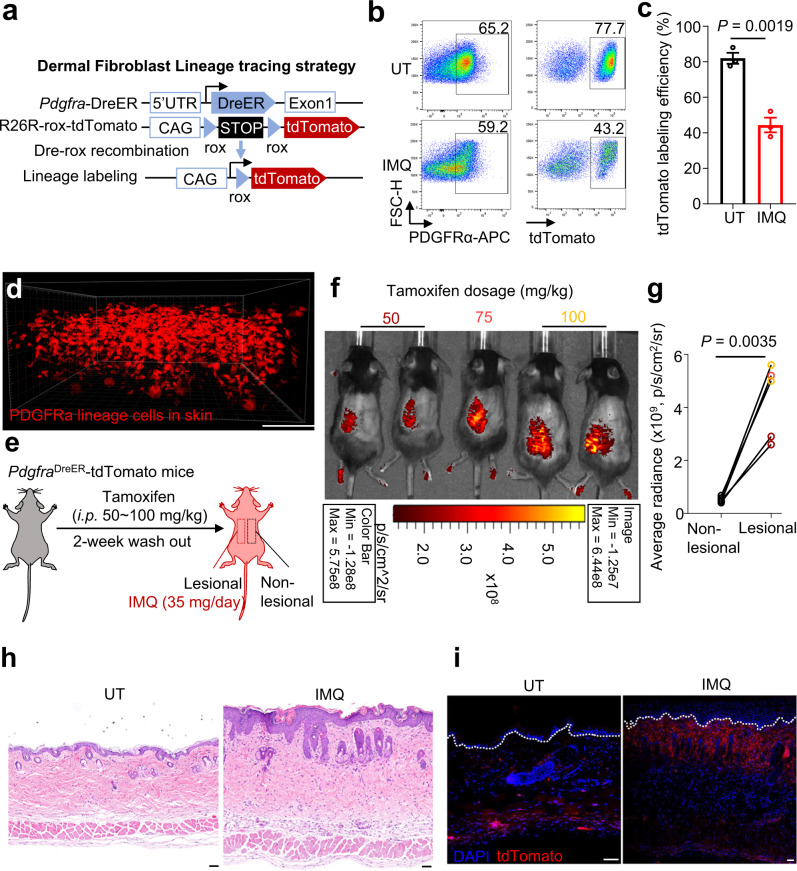
Lineage-tracing-based imaging shows altered fibroblast quantity and distribution in inflamed mouse skin. **a** Fibroblast lineage-tracing strategy of *Pdgfra*^DreER^-tdTomato mice. Flow cytometric analysis (**b**) and quantification (**c**) of tdTomato labeling efficiency in dermal PDGFRa^+^ fibroblasts of untreated (UT) and imiquimod (IMQ)-induced mouse skin (*n* = 3). **d** Representative whole-mount images of untreated *Pdgfra*^DreER^-tdTomato mouse skin. Scale bar, 100 μm**. e** Schematic diagram of IMQ-induced psoriasis mouse model in self-control *Pdgfra*^DreER^-tdTomato mice with different tamoxifen dosages. Luminescence imaging (**f**) and quantification (**g**) of tdTomato intensity in IMQ-induced self-control *Pdgfra*^DreER^-tdTomato mice (*n* = 5). Representative hematoxylin and eosin (H&E, **h**) and immunofluorescent (**i**) images of UT or IMQ-induced *Pdgfra*^DreER^-tdTomato mouse skin (*n* = 5). Scale bar, 50 μm. Data are representatives of two independent experiments. Data in (**c**) are presented as the mean ± SEM. The *P* values were calculated by two-tailed paired Student’s *t*-test. Source data are provided as a Source Data file.

*Pdgfra* is a robust marker for dermal fibroblasts and is expressed at different developmental stages^3^. We utilized *Pdgfra*^DreER^-tdTomato mice^23^ to label fibroblast lineage cells in the skin (Fig. 1a). Tamoxifen-induced Dre-rox recombination resulted in efficient tdTomato labeling and over 75% of dermal fibroblast lineage cells were labeled in untreated (UT) mouse skin (Fig. 1b, c), including those in the dermal papillae, dermal sheaths, and cutaneous adipose tissue (Fig. 1d). We then applied imiquimod (IMQ)^24^, a Toll-like receptor 7/8 agonist, to induce psoriasiform skin inflammation in the *Pdgfra*^DreER^-tdTomato mice (Fig. 1e). Luminescence imaging revealed a significant increase of tdTomato intensity in psoriasiform lesions, compared with UT skin (Fig. 1f, g), suggesting an expansion of the fibroblast lineage in response to inflammation. Densely packed cell nuclei (Fig. 1h) and intense tdTomato fluorescence appeared at the dermal-epidermal junction (DEJ) of the psoriasiform lesion (Fig. 1i), indicating altered distribution of dermal fibroblasts.

### A tenascin-C^+^ papillary fibroblast subset emerges in inflamed skin

**Fig. 2:**
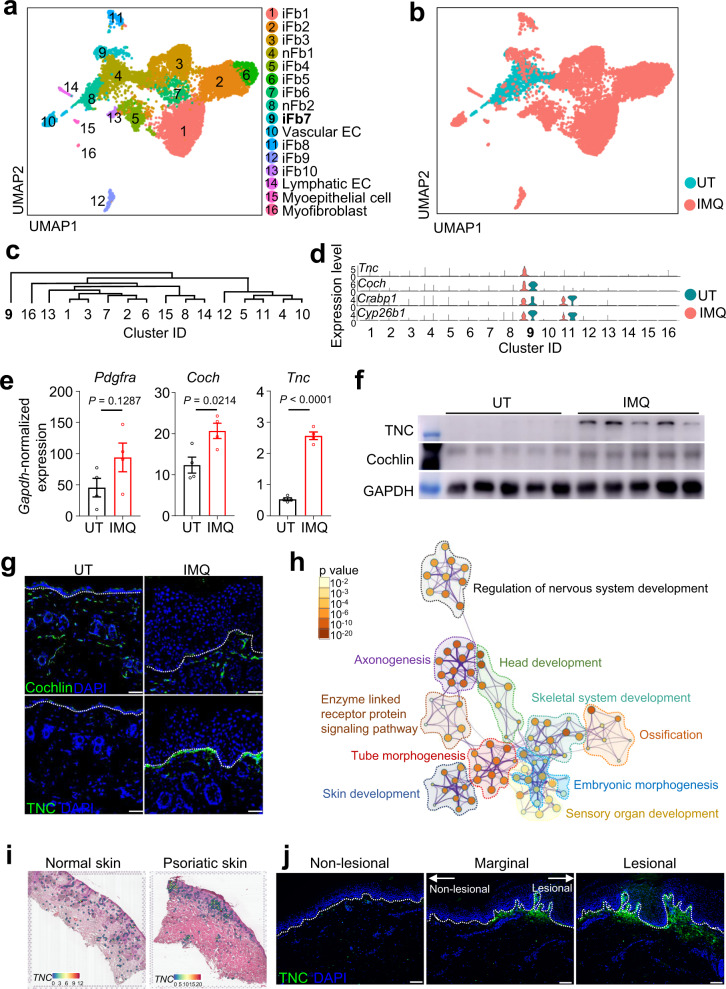
A tenascin-C (TNC)+ fibroblast subpopulation emerges at the dermal-epidermal junction (DEJ) of psoriatic skin. UMAP plot (**a, b**) and cluster dendrogram (**c**) of *Pdgfra*-lineage cells sorted from back skin of UT and IMQ-induced *Pdgfra*^DreER^-tdTomato mice. **d** Violin plots of cluster 9 signature genes expression. **e** qPCR analysis of *Pdgfra*, *Coch*, and *Tnc* in the dermis of UT or IMQ-induced mice (*n* = 4). **f** Immunoblot of Cochlin and TNC expression in the dermis of UT or IMQ-induced mice (*n* = 5). **g** Representative immunofluorescent images of Cochlin (upper panels) and TNC (lower panels) expression in UT or IMQ-induced mice (*n* = 5). Scale bar, 50 μm**. h** Top-ranked Gene Ontology (GO) pathways of *Coch*^+^*Tnc*^+^ fibroblast-enriched genes. **i** Representative spatial transcriptome images of *TNC* expression in normal (*n* = 2) or psoriatic human skin (*n* = 3). **j** Representative immunofluorescent images of TNC expression in non-lesional /marginal/lesional skin of human psoriatic skin (*n* = 5). Scale bar, 100 μm. Data are representatives of two independent experiments. Data in (**e**) are presented as the mean ± SEM. The *P* values were calculated by two-tailed unpaired Student’s *t*-test. Source data are provided as a Source Data file.

To understand fibroblast heterogeneity at the transcriptional level, we used single-cell RNA sequencing (scRNA-seq) to analyze CD45^-^tdTomato^+^ fibroblast lineages in UT and IMQ-induced *Pdgfra*^DreER^-tdTomato mouse skin (Supplementary Fig. 1a, b). Unbiased clustering of the integrated data resulted in 16 subpopulations defined by cell source and signature genes (Fig. 2a, b and Supplementary Fig. 1c–e). Clusters identified as being composed mainly of cells from IMQ-induced skin were designated induced fibroblast (iFb) clusters, while those consisting largely of cells from UT skin were named normal fibroblast (nFb) clusters. Vascular endothelial cell (EC), lymphatic EC, myoepithelial cell, and myofibroblast clusters were named according to their distinct gene characteristics. In general, IMQ-induced fibroblasts showed upregulated expression of adipogenic (*Plac8*, *Plin2*), pro-inflammatory (*Il6*, *Cd44*), and chemotactic (*Ccl2*, *Ccl7*, *Cxcl1*, *Cxcl12*) genes (Supplementary Fig. 1f), and demonstrated a remarkable skewing towards lineages adapted to inflammation (Fig. 2b and Supplementary Fig. 1c).

The dendrogram showed that cluster 9 (iFb7), marked by *Coch* and *Tnc* (Fig. 2d), represented a distinct cluster with a unique transcriptional profile (Fig. 2c). We verified that both *Coch* and *Tnc* were significantly upregulated at the transcriptional and translational level in IMQ-induced mouse skin, compared with UT skin (Fig. 2e, f). Notably, the *Tnc*-encoded protein tenascin-C (TNC) was barely expressed under homeostatic conditions. However, immunostaining revealed high levels of TNC at the DEJ of IMQ-induced mouse skin (Fig. 2g), suggesting that *Tnc* expression was stimulus-responsive.

To understand the biological functions of the *Coch*^+^*Tnc*^+^ fibroblasts, we performed Gene Ontology (GO) pathway analysis of the co-regulated genes of this subpopulation (Supplementary Fig. 2a–c). Pathways indicating axonogenesis, regulation of nervous system development, and skin development were significantly enriched, suggesting a neuromodulation function for these fibroblasts during skin inflammation (Fig. 2h). Analysis of human scRNA-seq data (GSE150672)^25^ revealed a small fraction of *COCH*^+^*TNC*^+^*TNN*^+^ fibroblasts showing similar GO pathway enrichment (Supplementary Fig. 2d). *TNC* showed upregulated expression level in human psoriatic skin fibroblasts compared with normal fibroblasts (Supplementary Fig. 2e, f). Spatial transcriptome analysis of human skin samples showed that *TNC* was rarely transcribed in normal or non-lesional skin but was significantly induced at the DEJ of psoriatic lesions (Fig. 2i). In human psoriatic skin with both lesional and non-lesional regions (Supplementary Fig. 2g), TNC showed escalating staining intensity from non-lesional to lesional area (Fig. 2j). Together, these data identified a subset of TNC-secreting papillary fibroblasts located at the DEJ in psoriatic mouse and human skin and exhibiting neuroregulatory features.

### Inflammation rearranges dermal fibroblast and cutaneous nerve histology

**Fig. 3:**
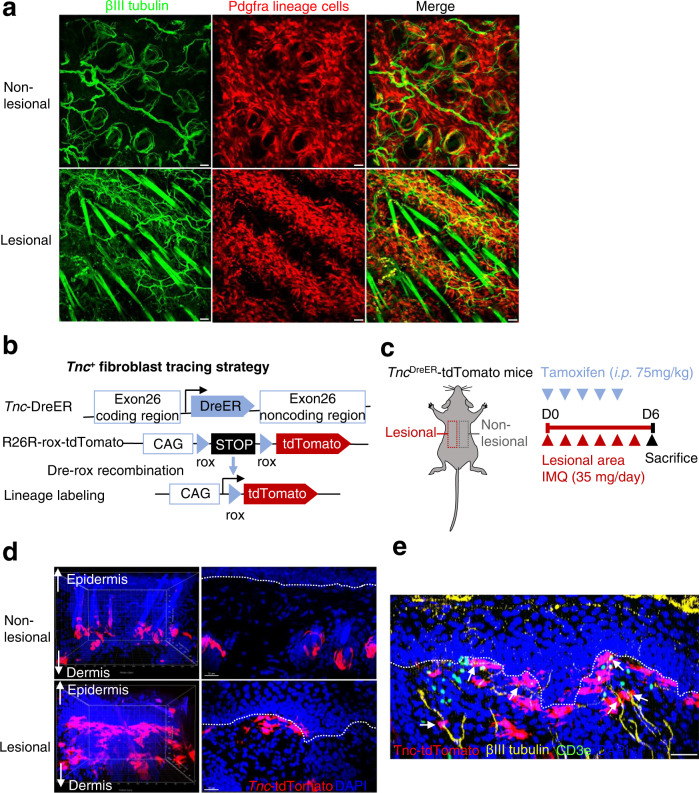
Papillary fibroblasts interacted with cutaneous nerves in the inflamed skin. **a** Representative whole-mount images of non-lesional or lesional skin stained for βIII tubulin (green) of IMQ-induced self-control *Pdgfra*^DreER^-tdTomato mice (*n* = 3). Scale bar, 50 μm. **b** Tracing strategy of *Tnc*^DreER^-tdTomato mice. **c** Schematic diagram of IMQ-induced psoriasis mouse model in self-control *Tnc*^DreER^-tdTomato mice. **d** Representative whole-mount images of non-lesional or lesional skin of self-control *Tnc*^DreER^-tdTomato mice in (**c**, *n* = 3). Scale bar, 15 μm. **e** Representative whole-mount images of lesional skin stained for βIII tubulin (yellow), and CD3e (green) of *Tnc*^DreER^-tdTomato mouse lesional skin in (**c** *n* = 3). Scale bar, 50 μm. Data are representatives of two or three independent experiments.

Whole-mount imaging of *Pdgfra*^DreER^-tdTomato mice demonstrated a pattern of loosely arranged fibroblasts in non-lesional skin; which contrasted with the dense arrangement of fibroblasts and increased numbers of interwound nerve fibers observed in the lesional papillary dermis (Fig. 3a). Given the distinct expression pattern of TNC in the papillary dermis, we then constructed *Tnc*^DreER^-tdTomato mice in order to observe *Tnc*^+^ fibroblast location (Fig. 3b). Adult *Tnc*^DreER^-tdTomato mice were subjected to a “self-control” IMQ-induced mouse model whereby half of the back skin was treated with IMQ (lesional) and half was left untreated (non-lesional) to reduce the effect of individual differences in responses (Fig. 3c). Whole-mount images showed that *Tnc*^+^ fibroblasts were restricted to hair follicles in non-lesional skin but emerged in the papillary dermis upon IMQ stimuli (Fig. 3d). Notably, tdTomato fluorescence signals produced by *Tnc*^+^ fibroblasts were detected adjacent to βIII tubulin^+^ nerve fibers and CD3e^+^ T cells in the lesional papillary dermis (Fig. 3e).

### *Tnc*^+^ fibroblasts promote axonogenesis

**Fig. 4:**
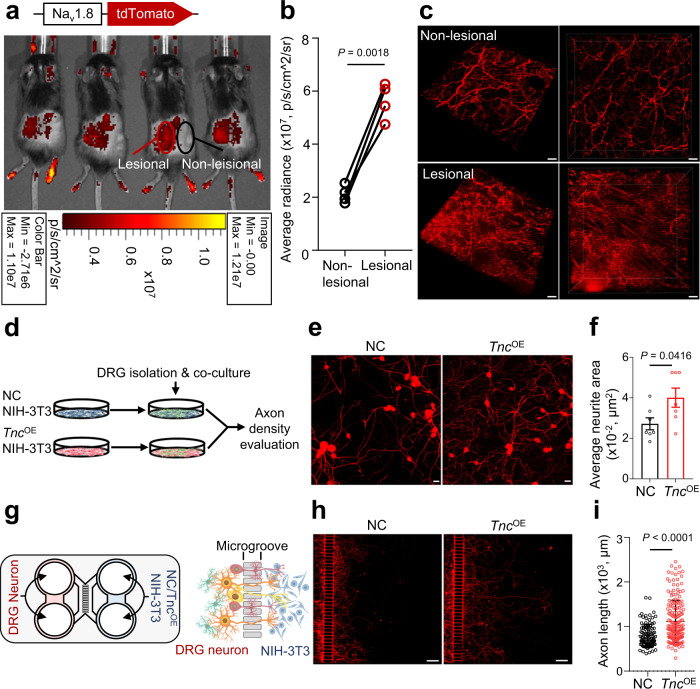
TNC promotes aberrant neurite outgrowth. Luminescence imaging (**a**) and quantification (**b**) of tdTomato^+^ cutaneous nerves in self-control IMQ-induced Na_v_1.8-tdTomato reporter mice (*n* = 4). **c** Representative whole-mount images of non-lesional or lesional skin in self-control IMQ-induced Na_v_1.8-tdTomato reporter mice (*n* = 4). Scale bar, 50 μm. **d** Schematic diagram of dorsal root ganglion (DRG) neuron and NC/*Tnc*^OE^ NIH-3T3 cell direct co-culture assay. NC negative control. *Tnc*^OE^, *Tnc*-overexpressing. Representative immunofluorescent images (**e**) and quantification (**f**) of average neurite area per cell (stained for βIII tubulin, red) in the DRG neuron and NC/*Tnc*^OE^ NIH-3T3 cell direct co-culture dishes (*n* = 7). Scale bar in (**e**), 50 μm**. g** Schematic diagram of DRG neuron and NC/*Tnc*^OE^ NIH-3T3 cell co-culture chamber assay. Representative immunofluorescent images (**h**) and quantification (**i**) of neurite length (stained for βIII tubulin, red) in the DRG neuron and NC/*Tnc*^OE^ NIH-3T3 cell co-culture chambers. Scale bar in (**h**), 200 μm. Neurite lengths over 300 μm were calculated in (**i**, *n* = 103 for NC group and *n* = 188 for *Tnc*^OE^ group). Data are representatives of three independent experiments. Data in (**f, i**) are presented as the mean ± SEM. The *P* values were calculated by two-tailed paired (**b**) or unpaired (**f**, **i**) Student’s *t*-test. Source data are provided as a Source Data file.

Given the neuroregulatory transcriptional features of *Tnc*^+^ fibroblasts, we subjected Na_v_1.8-tdTomato peripheral sensory nerve reporter mice to the IMQ-induced psoriasis model. Comparison of lesional and non-lesional skin revealed significantly stronger tdTomato signal intensity in lesional skin, suggesting hyper-innervated histology in psoriasiform skin (Fig. 4a, b). Whole-mount imaging demonstrated hyperinnervation with anomalous bifurcation in psoriasiform lesions (Fig. 4c).

Sensory nerve blockade or denervation of nociceptors has been reported to relieve psoriasis by inhibiting IL-23/type 17 responses^14,18,19^, highlighting the crucial role of cutaneous nerves in skin inflammation. To investigate whether inflammation-induced TNC accounts for the aberrant neurite outgrowth in mouse psoriasiform lesions, we overexpressed full-length mouse *Tnc* in the fibroblast cell line NIH-3T3 (Supplementary Fig. 3a, b) and performed a dorsal root ganglia (DRG) neuron-fibroblast co-culture assay (Fig. 4d). Mouse DRG neurons co-cultured with *Tnc-*overexpressing (*Tnc*^OE^) fibroblasts exhibited denser array of neurites than those co-cultured with negative control (NC) fibroblasts (Fig. 4e, f). Skin is innervated by sensory neuron terminals, while the cell bodies are found in the trigeminal and DRG^26,27^. To simulate this pattern, we co-cultured DRG neurons with NC/*Tnc*^OE^ fibroblasts in chambers where the neuron cell bodies were separated from the axon terminals (Fig. 4g). Notably, increased axon length was observed in chambers seeded with monolayers of *Tnc*^OE^ fibroblasts rather than NC cells, indicating that *Tnc*^OE^ fibroblasts were able to promote axonogenesis at peripheral sites in the absence of contact with neuronal somas (Fig. 4h, i).

Several integrins have been identified as TNC receptors on neuronal membranes and involved in sensory neurite outgrowth^28–30^. Among these integrins, online database (GSE131230)^31^ analysis revealed that α7β1 was predominately expressed by DRG nociceptors (Supplementary Fig. 4a). Besides, α7β1 subunits were significantly upregulated at the transcriptional level in IMQ-induced mouse primary DRG neurons compared with that of UT mice (Supplementary Fig. 4b), indicating they may participate in inflammation-induced neuropathy. ERK signaling is pivotal in integrin-mediated neurite outgrowth^32,33^. Our results showed that recombinant TNC promoted neurite outgrowth and ERK1/2 phosphorylation in a dose-dependent manner (Supplementary Fig. 4c, d). More importantly, ERK agonist butylhydroquinone (TBHQ) further promoted TNC-induced neurite anomalous bifurcation, while ERK inhibitor AZD6244 strongly suppressed neurite outgrowth in the presence of TNC (Supplementary Fig. 4e). Taken together, these results suggested that the pro-axonogenesis capacity of TNC is ERK signaling-dependent.

### Injury/inflammation-induced TNC exacerbates skin inflammation

**Fig. 5:**
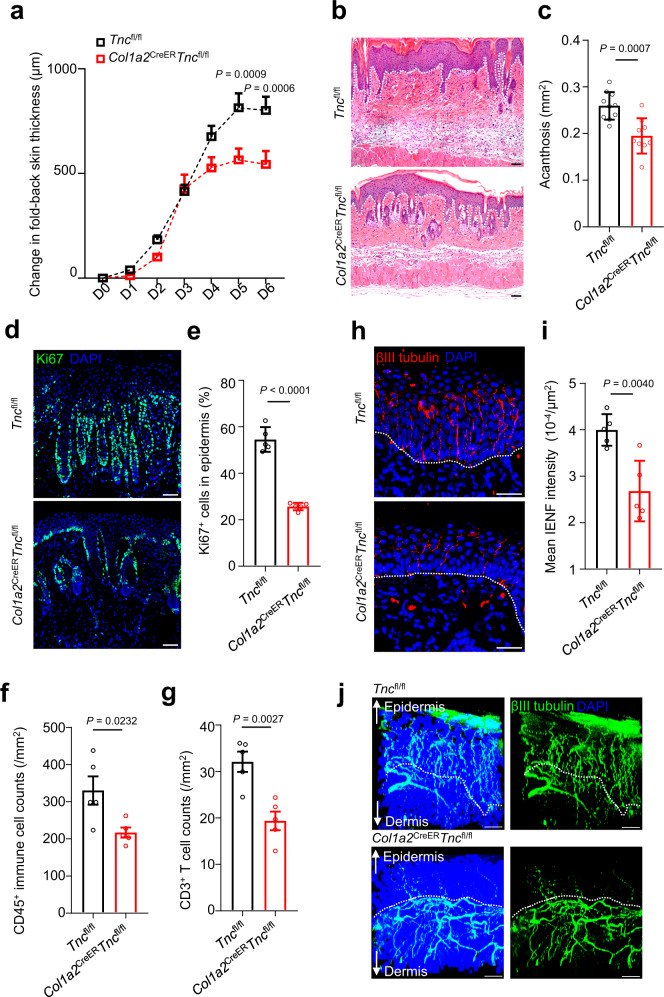
Fibroblast-specific ablation of *Tnc* restrains skin inflammation and excessive axonogenesis in mouse psoriasiform lesions. **a** Change in fold-back skin thickness of IMQ-induced *Tnc*^fl/fl^ (male: *n* = 5; female: *n* = 5) or *Col1a2*^CreER^*Tnc*^fl/fl^ (male: *n* = 5; female: *n* = 4) mice. Representative H&E staining images (**b**) and quantification of skin acanthosis (**c**) of IMQ-induced *Tnc*^fl/fl^ (male: *n* = 5; female: *n* = 5) or *Col1a2*^CreER^*Tnc*^fl/fl^ mice (male: *n* = 5; female: *n* = 4). Scale bar in (**b**), 50 μm. Representative immunofluorescent images (**d**) and quantification (**e**) of Ki67^+^ cells in the epidermis of IMQ-induced *Tnc*^fl/fl^ or *Col1a2*^CreER^*Tnc*^fl/fl^ mice (*n* = 5). Scale bar in (**d**), 50 μm. Quantification of CD45^+^ immune cells (**f**) and CD3^+^ T cells (**g**) in the skin of IMQ-induced *Tnc*^fl/fl^ or *Col1a2*^CreER^*Tnc*^fl/fl^ mice (*n* = 5). Representative immunofluorescent images (**h**) and quantification (**i**) of intra-epidermal nerve fiber (IENF) intensity in the skin of IMQ-induced *Tnc*^fl/fl^ or *Col1a2*^CreER^*Tnc*^fl/fl^ mice (*n* = 5). Scale bar in (**h**), 50 μm. **j** Representative whole-mount images of nerve fibers in the skin of IMQ-induced *Tnc*^fl/fl^ or *Col1a2*^CreER^*Tnc*^fl/fl^ mice (*n* = 3). Scale bar, 30 μm. Data are representatives of two independent experiments and presented as mean ± SEM. The *P* values were calculated by two-way ANOVA and Holm–Šídák test (**a**) or two-tailed unpaired Student’s *t*-test (**c**, **e**, **f**, **g**, **i**). Source data are provided as a Source Data file.

To investigate whether TNC contributes to psoriasiform skin inflammation, we generated *Col1a2*^CreER^*Tnc*^fl/fl^ mice with specific ablation of TNC in fibroblasts and evaluated the severity of IMQ-induced skin inflammation (Supplementary Fig. 3c, d). *Col1a2*^CreER^*Tnc*^fl/fl^ mice showed significant relief of psoriasiform skin inflammation compared with *Tnc*^fl/fl^ mice, as indicated by skin thickness (Fig. 5a), acanthosis (Fig. 5b, c), percentage of Ki67^+^ proliferating cells in the epidermis (Fig. 5d, e), and CD45^+^ immune cell and CD3^+^ T-cell infiltration (Fig. 5f, g). Meanwhile, fibroblast-conditional TNC knockout rescued hyperinnervation in psoriatic lesions as shown by intra-epidermal nerve fiber immunostaining (Fig. 5h, i) and whole-mount cutaneous nerve imaging (Fig. 5j). Collectively, these results showed that TNC deficiency in fibroblasts suppressed mouse psoriasiform skin inflammation and restrained excessive axonogenesis.

The expression of TNC is subtly regulated, being absent in normal tissues but prominent at sites of inflammation, trauma, and solid tumor stroma^34–38^. In the clinic, patients with psoriasis and other skin diseases are susceptible to the Koebner phenomenon, whereby new psoriatic lesions appear on previously unaffected skin as a secondary effect of trauma^39,40^. Tape stripping (TS) is a common way to induce mild cutaneous injury^41,42^ and is sufficient to drive Koebner lesion formation in transgenic mice that spontaneously develop psoriasis^43^. We designed a TS-pre-treated psoriasis mouse model to assess TNC expression following skin injury and its correlation with skin inflammation severity (Supplementary Fig. 5a). In this model, TS destroyed the skin barrier and increased trans-epidermal water loss (TEWL, Supplementary Fig. 5b). Pre-tape-stripped skin showed more severe skin inflammation following IMQ administration than skin that had not been subjected to TS, as measured by TEWL scores and skin acanthosis (Supplementary Fig. 5b–d). Immunoblotting results showed that TS alone led to a slight increase in TNC expression, while combining TS with IMQ stimulation further increased levels of TNC in the skin (Supplementary Fig. 5e). Notably, we found a positive correlation between skin acanthosis severity and TNC expression (Supplementary Fig. 5f), as well as between the expression of neuronal marker βIII tubulin and TNC (Supplementary Fig. 5g). These results demonstrated that TNC could be induced by trauma, while its expression level correlated with skin neurite content and inflammation severity.

### TNC aggravates skin inflammation through peripheral nociceptors

**Fig. 6:**
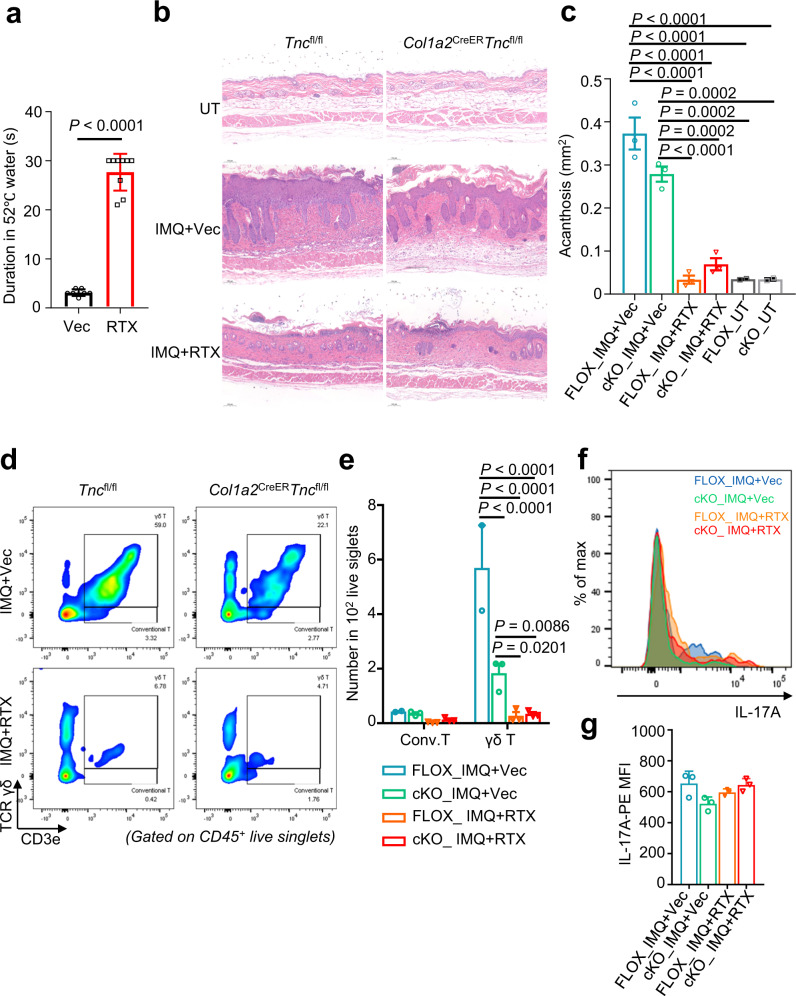
Cutaneous nociceptors serve as essential downstream of TNC in mediating skin inflammation. **a** Quantification of tail flick time in 52 °C hot water of vehicle (Vec, *n* = 7) or resiniferatoxin (RTX, *n* = 9)-treated *Tnc*^fl/fl^ and *Col1a2*^CreER^*Tnc*^fl/fl^ mice. Representative H&E staining images (**b**) and quantification (**c**) of skin acanthosis of untreated (UT)/IMQ-induced *Tnc*^fl/fl^ or *Col1a2*^CreER^*Tnc*^fl/fl^ mice treated with Vec or RTX (*n* = 2–3 per group). FLOX, *Tnc*^fl/fl^; cKO, *Col1a2*^CreER^*Tnc*^fl/fl^. Scale bar in (**b**), 100 μm. Flow cytometric analysis (**d**) and quantification (**e**) of conventional T-cell and γδT cells in skin lesions of IMQ-induced *Tnc*^fl/fl^ or *Col1a2*^CreER^*Tnc*^fl/fl^ mice treated with Vec or RTX (*n* = 2–3 per group). Flow cytometric analysis (**f**) and quantification (**g**) of IL-17A expression in T cells in psoriasiform skin lesions of *Tnc*^fl/fl^ or *Col1a2*^CreER^*Tnc*^fl/fl^ mice treated with Vec or RTX (*n* = 3 per group). Data are representatives of two independent experiments and presented as mean ± SEM. The *P* values were calculated by two-tailed unpaired Student’s *t*-test (**a**), two-way ANOVA and Holm–Šídák test (**c**, **e**) and one-way ANOVA and Tukey’s test (**g**). Source data are provided as a Source Data file.

To evaluate whether TNC aggravates psoriasiform skin inflammation through cutaneous nerves, we specifically ablated TRPV1-expressing peripheral nociceptors with resiniferatoxin (RTX)^14,20^ prior to IMQ application. RTX treatment blocked tail nociception to noxious heat stimuli (Fig. 6a), and strongly suppressed IMQ-induced skin acanthosis in both *Tnc*^fl/fl^ and *Col1a2*^CreER^*Tnc*^fl/fl^ mice. Notably, exfoliation was commonly seen after denervation, suggesting the role of peripheral nerves in regulating epidermal integrity and function (Fig. 6b, c). Fibroblast-conditional TNC knockout significantly decreased CD3e^+^ T-cell especially γδT-cell infiltration compared with *Tnc*^fl/fl^ mice. Denervation dramatically reduced recruited γδT cells in skin lesions, which are comparable between denervated *Tnc*^fl/fl^ and *Col1a2*^CreER^*Tnc*^fl/fl^ mice (Fig. 6d, e). The IL-17A-producing capacity of γδT cells in *Col1a2*^CreER^*Tnc*^fl/fl^ mouse skin lesions slightly decreased but showed no significance compared with *Tnc*^fl/fl^ mice. Meanwhile, little effect of denervation on γδT-cell IL-17A production was observed (Fig. 6f, g). These results demonstrated that peripheral nociceptive sensory neurons served as pivotal downstream of TNC and upstream of epidermal hyperplasia and immune cell infiltration during psoriasiform skin inflammation.

### Postsynaptic signaling pathways regulate pro-inflammatory dermal γδT cells

**Fig. 7:**
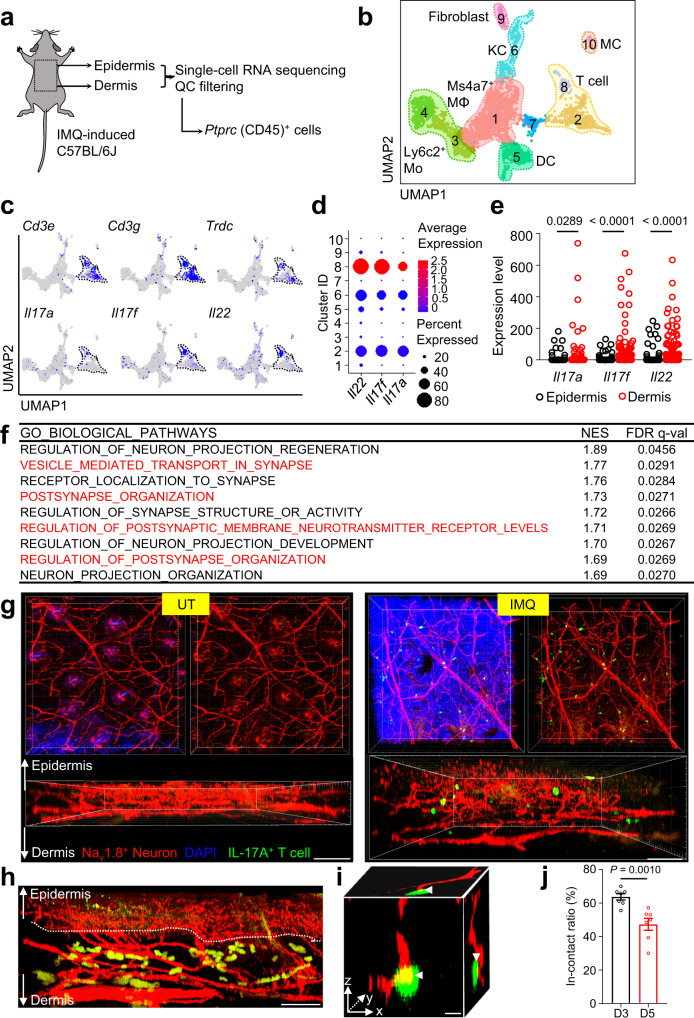
Pathogenic γδT cells are in contact with cutaneous nerves and enriched in postsynaptic pathways. **a** Workflow of scRNA-seq and data extraction for *Ptprc*^+^ (CD45^+^) immune cells in IMQ-induced mouse skin. **b** UMAP plot of immune cell clustering in IMQ-induced mouse skin. **c** Feature plots of pathogenic T-cell subset signature genes. **d** Dot plot of *Il22*, *Il17f*, and *Il17a* expression by clusters in (**b**). **e** Comparison of *Il22*, *Il17f*, and *Il17a* expression between epidermal and dermal T cells. Each point represents a single T cell (*n* = 352 for epidermis T-cell group and n = 331 for dermis T-cell group). **f** Enriched GO biological pathways of upregulated differentially expressed genes in *Il17a*^+^ versus *Il17a*^-^ dermal T cells. **g** Representative whole-mount images of Na_v_1.8^tdTomato^IL-17A^EGFP^ dual reporter mouse skin with no treatment (UT, left panels) or 5-day IMQ administration (right panels, *n* = 3 mice per group). Scale bar, 100 μm. **h** Representative whole-mount image of Na_v_1.8^tdTomato^IL-17A^EGFP^ dual reporter mouse skin with 3-day IMQ administration (*n* = 3 mice per group). IL-17A^+^ T cells in contact with cutaneous nerves in the upper dermis are marked with white arrows. Scale bar, 50 μm. **i** Representative image of a 3-dimensional interaction between IL-17A^+^ T cell (EGFP, green) and cutaneous nerves (tdTomato, red). The interaction sites are indicated by white arrowheads. Scale bar, 30 μm. **j** Quantification of in-contact ratio between IL-17A^+^ T cell and cutaneous nerves in psoriasiform mouse skin with 3-day or 5-day IMQ administration (*n* = 7 mouse skin samples per group). Data in (**g**–**j**) are representative of three independent experiments. Data in (**j**) are presented as the mean ± SEM. The *P* values were calculated by two-tailed unpaired Student’s *t*-test. Source data are provided as a Source Data file.

The IL-23/IL-17 immune axis is the dominant pathway that drives psoriasis pathology^11,13^. To understand how cutaneous nerve alterations regulate immune responses in mouse psoriasiform lesions, scRNA-seq analysis of CD45^+^ (encoded by *Ptprc*) immune cells in IMQ-induced mouse skin was performed (Fig. 7a). In accordance with previous studies^44,45^, our data demonstrated that γδT cells (Fig. 7b–d and Supplementary Fig. 6a, b), especially those in the dermis (Fig. 7e), were the primary source of type 17 pathogenic cytokines (*Il17a*, *Il17f*, and *Il22*). Furthermore, GO analysis showed that *Il17a*^+^ dermal T cells exhibited enrichment of postsynaptic pathways, relative to *Il17a*^−^ counterparts (Fig. 7f), suggesting that they were regulated by neuronal signals during skin inflammation.

Several studies have revealed a type 17 immune response in skin following nociceptive sensory neuron activation^14,20^. However, direct interaction between pathogenic T cells and cutaneous nerves has not been demonstrated. Using Na_v_1.8^tdTomato^IL-17A^EGFP^ dual reporter mice, we visualized the spatial interaction between IL-17A^+^ T cells and cutaneous sensory nerves in mouse psoriasiform lesions. Numbers of IL-17A-EGFP^+^ T cells significantly increased as early as 3 days post-IMQ administration and further expanded on day 5, while EGFP signal was not detected in the skin of UT mice (Fig. 7g). The majority of IL-17A-EGFP^+^ T cells appeared in the papillary dermis on day 3 (Fig. 7h) and showed dispersive distribution on day 5 (Fig. 7g, right panel). Cutaneous nerves and T cells were considered to be in contact when tdTomato and EGFP signals converged in the 3-dimensional space (Fig. 7i and Supplementary Fig. 7). The in-contact ratio reached 50–60% on day 3 and declined slightly on day 5 in mouse psoriasiform lesions (Fig. 7j). To summarize, pro-inflammatory γδT cells were in spatial contact with cutaneous sensory nerves at an early phase of skin inflammation and were likely regulated by neuronal signals via neuro-immune synapses.

## Discussion

Hyperinnervation accompanied by elevated neurotransmitter levels is common in psoriatic lesions of patients and rodents^15,46^ and vital for local immunity driven by cutaneous nerves^14,18–20^. However, little is known about the local signals that promote innervation during skin inflammation. Here, we identified a subset of TNC-secreting papillary fibroblasts that emerged at the DEJ following skin irritation and promoted aberrant neurite outgrowth, facilitating the formation of pathogenic neuro-immune synapses and exacerbating skin inflammation (Supplementary Fig. 8).

TNC has been reported to be expressed throughout the skin basement membrane in psoriasis and several other hyperproliferative skin diseases, remaining abundant after clinical lesion remission;^34,37,47^ with its functions uncharacterized. Through lineage-tracing-based scRNA-seq and imaging, we identified that TNC is strictly secreted by a subset of inflammation-induced papillary fibroblasts with pro-axonogenesis transcriptional hallmarks. *Tnc*^OE^ fibroblasts boosted neurite outgrowth of co-cultured neurons; and fibroblast-specific *Tnc* ablation in mice showed diminished TNC expression in the lesional papillary dermis, along with restrained cutaneous innervation and alleviative skin inflammation. We found *Tnc* itself defined a more specific pro-inflammatory fibroblast subset which shares similar localization and gene profiles with previous identified inflammatory dermal fibroblast subsets^6,48^. TNC is highly conserved amongst vertebrates^49,50^. The fibronectin type-III repeat domain of TNC promotes sensory axon regeneration^51,52^, while several integrins have been identified as TNC receptors on neuronal membranes^28–30^. Our results revealed that integrin α7β1 was strongly induced in DRG neurons upon IMQ stimulation, providing empirical evidence for interactions between *Tnc*^+^ fibroblasts and cutaneous nerves. Taken together, this inflammation-induced TNC^+^ papillary fibroblast subset may exert prolonged effects in psoriatic lesions through extracellular matrix remodeling and remain a risky niche for lesion recurrence.

The type 17 inflammatory response elicited by cutaneous sensory nerve activation has been attributed to the contact with dermal DCs, which further activate T17 cells through IL-23 secretion and initiate psoriatic lesion formation^14,20^. Dermal γδT cells, the pivotal source of type 17 cytokines in psoriasis, exhibit effector memory cell characteristics and are poised to produce IL-17 rapidly in the periphery^53,54^. Our results have provided further clues for pathogenic γδT-cell IL-17 production, showing that it may be directly regulated by neuronal signals; however, the precise mechanisms remain to be clarified. While sustained interaction and synapse formation between type 17 T cells and nerve fibers have been reported^55,56^, super-resolution in vivo imaging and spatial proteomics may help us to understand the delicate structure of neuro-immune synapses and identify the relevant transmitters.

In summary, our study identified an inflammation-induced *Tnc*^+^ papillary fibroblast subset, illuminating the stroma–neuron interactions underlying psoriasiform skin inflammation. Given their role in promoting axonogenesis and scaffolding neuro-immune synapses, *Tnc*^+^ fibroblasts and TNC may represent novel targets for the treatment of psoriasis and other inflammatory skin diseases.

## Methods

### Human subjects

Analysis studies involving human skin biopsies were reviewed and approved by the Ethics Committee of Shanghai General Hospital, China (No. 2018KY239). All donors provided written informed consent. For spatial transcriptomics analysis, skin biopsies were obtained from treatment-naïve patients with psoriasis and control healthy donors who had surgeries, operations were performed by the principles of the Declaration of Helsinki. For histologic and immunofluorescent imaging, paraffin sections of psoriatic lesions with the adjacent non-lesional region were provided by Dr. Z. Yao (Xinhua Hospital, Shanghai Jiao Tong University School of Medicine).

### Mice

C57BL/6 mice, 6–8 weeks old, were purchased from Shanghai SLAC Laboratory Animal Co., Ltd (Shanghai, China). *Col1a2*-CreER^57^, *Pdgfra*-DreER^23^, R26R-rox-tdTomato^58^, R26R-tdTomato^59^ mouse line were previously described. Na_v_1.8-Cre mouse line^60^ was provided by Dr. Xiaoyang Cheng (Department of Anatomy, Histology, and Embryology, Shanghai Jiao Tong University School of Medicine, China). IL-17-GFP mouse line (MGI: J:184819) was provided by Dr. Zhinan Yin (The First Affiliated Hospital, Biomedical Translational Research Institute and Guangdong Province Key Laboratory of Molecular Immunology and Antibody Engineering, Jinan University, Guangzhou, China). *Tnc*-DreER was generated by homologous recombination using CRISPR-Cas9 technology. In short, a cDNA encoding P2A-DreER-WPRE-polyA was inserted after coding region of *Tnc* gene before 3′UTR. *Tnc*^fl/fl^ mouse line was purchase from Cyagen Biosciences Inc (cat: CKOCMP-21923-Tnc-B6J-VA). *Pdgfra*^DreER^-tdTomato and *Tnc*^DreER^-tdTomato mice were generated by crossing the *Pdgfra*^DreER^ or *Tnc*^DreER^ with the R26R-rox-tdTomato reporter line, respectively. Na_v_1.8-tdTomato mice were generated by crossing the Na_v_1.8-Cre with the R26R-tdTomato reporter line, and were further crossed with the IL-17-GFP line to obtain the Na_v_1.8^tdTomato^IL-17^GFP^ dual reporter mice. *Col1a2*^CreER^*Tnc*^fl/fl^ mice were generated by crossing the *Col1a2*-CreER with the *Tnc*^fl/fl^ mouse line. Tamoxifen (Sigma, T5648) was dissolved in corn oil and injected intraperitoneally at indicated dosage. For *Pdgfra*^DreER^-tdTomato, *Col1a2*^CreER^*Tnc*^fl/fl^ and *Tnc*^fl/fl^ mice, tamoxifen was administered at 5-week-old for 5 consecutive days, and psoriasis mouse model was performed after 2-week washout period. For *Tnc*^DreER^-tdTomato mice, tamoxifen was simultaneously administered with IMQ to induce efficient labeling. The mice were bred and maintained under specific pathogen-free (SPF) conditions. Mice were housed in cages with five mice per cage and kept on in a regular 12 h:12 h light:dark cycle. The temperature was 22 ± 1 °C and humidity was 40–70%. Age-matched and sex-matched mice were used for all of the experiments approved by the National Institutes of Health Guide for the Care and Use of Laboratory Animals with the approval (SYXK-2003-0026) of the Scientific Investigation Board of Shanghai Jiao Tong University School of Medicine in Shanghai, China. To ameliorate any suffering that the mice observed throughout these experimental studies, the mice were euthanized by CO_2_ inhalation.

### Cell culture

NIH-3T3 cells (ATCC, cat: CRL-1658) were cultured in DMEM/high glucose (HyClone, cat: SH30022.01) containing 10% fetal bovine serum (FBS, Gibco, cat: 10099141C). The medium was refreshed every 2 days and the cells were sub-cultured according to the cell fusion. Cells in passages 2–6 were used for subsequent experiments. For stable *Tnc*^OE^ NIH-3T3 cell line construction, full-length cDNA encoding mouse *Tnc* was amplified from PCR-BluntII-TOPO plasmid (DNA Core of Shanghai Jiao Tong University School of Medicine) and then was inserted into the pReceiver-Lv215 vector (GeneCopreia^TM^) to obtain *Tnc*-Lv215 overexpressing plasmid. Lentivirus particles were obtained by co-transfecting *Tnc*-Lv215 with packaging plasmids (pMD2.G and psPAX2) into HEK293-FT cells (Invitrogen, cat: R70007). NIH-3T3 cells were infected with concentrated *Tnc*-Lv215 lentivirus particles and then were sorted for GFP^+^ cells to acquire a stable *Tnc*^OE^ NIH-3T3 cell line.

### IMQ-induced mouse model of psoriasis

Seven-week-old *Pdgfra*^DreER^-tdTomato mice, *Tnc*^DreER^-tdTomato mice*, Col1a2*^CreER^Tnc^*fl/fl*^ mice, Tnc^*fl/fl*^ mice, and wild-type male C57BL/6 mice were maintained under SPF conditions and subjected to IMQ-induced mouse model. Male mice were used if no otherwise noted. Both male and female *Col1a2*^CreER^Tnc^*fl/fl*^ and Tnc^*fl/fl*^ mice were used to test the effect of *Tnc* conditional knockout in IMQ-induced psoriasiform skin inflammation. To induce psoriasiform dermatitis, the mice were shaved and topically applied with 5% IMQ cream (MedShine, cat: 120503) for 6 consecutive days unless otherwise noted (62.5 mg for whole back skin, 35 mg for halfback skin, and 25 mg for each ear). On the indicated days, the skin thickness was measured with a micrometer (Schieblehre). For nociceptive sensory neuron denervation, RTX was injected subcutaneously into the back in three escalating doses (30 mg kg^−1^, 70 mg kg^−1^ and 100 mg kg^−1^) on consecutive days, and mice were allowed to rest for at least 4 weeks before IMQ treatment. For cutaneous injury, mouse shaved back skin was tape-stripped five times with Tegaderm (3 M™) for four consecutive days before IMQ administration. TEWL measurements were measured at room temperature (RT) and a humidity level of 50 ± 20% using a gpskin™ Barrier Light skin barrier function measurement device.

### Single-cell RNA sequencing (scRNA-seq)

Fresh skin was placed in saline at 4 °C until further processing. The epidermis was separated from the dermis by Dispase II (Roche, cat: 04942078001) digestion overnight at 4 °C. Single-cell suspensions were generated by enzyme digestion and subjected to fluorescence-activated cell sorting (FACS) to exclude doublets, debris, and DAPI^+^ dead cells. For *Pdgfra*-lineage cells scRNA-seq, dermal CD45^-^tdTomato^+^ live cells were sorted from untreated or 7-day-IMQ-induced *Pdgfra*^DreER^-tdTomato mouse skin; for immune cell scRNA-seq of the psoriasiform lesion, all epidermal live cells and dermal CD45^+^ live cells were sorted separately from 5-day-IMQ-induced C57BL/6 mouse skin. Sorted cells were centrifuged and resuspended in PBS containing 0.04% BSA. Chromium Single Cell 3′ v3 (10x Genomics) library preparation was conducted by the Sequencing Core at the Shanghai Institute of Immunology, according to the manufacturer’s instructions. The resulting libraries were sequenced with an Illumina HiSeq 4000 platform. Trimmed data were processed using the CellRanger (10x Genomics, version 3.0) and further filtered, processed, and analyzed using the Seurat package (version 3.1.2)^61^ with R (version 3.6.3).

### Data processing and clustering with Seurat

Cells with fewer than 200 genes, more than 5000 genes, or more than 5% mitochondria content were removed. Doublets were predicted using DoubletFinder and removed^62^. The filtered data were normalized using a scaling factor of 10,000 to generate transcripts per kilobase million (TPM)-like values. Filtered samples were integrated using the FindIntegrationAnchors and IntegrateData functions with default parameters (dimensionality = 30). For the data shown in Fig. 2, the UT dataset of 1340 cells and IMQ dataset of 9312 cells were integrated; for the data shown in Fig. 7, epidermal and dermal datasets were extracted for *Ptprc*^+^ (CD45^+^) immune cells separately (632 cells for epidermal dataset and 3203 cells for dermal dataset), and then integrated for further analysis. The top 2000 most variable genes were selected using the FindVariableFeatures function and the genes were then used for principal component analysis (PCA). The number of PCs for clustering was selected based on the ‘Elbow plot’ of different datasets, and clustering was performed using the FindClusters function with a resolution selected for different datasets as described below. For the data are shown in Fig. 2, 20 PCs and a resolution of 0.5 were used. For the data are shown in Fig. 7, 20 PCs and a resolution of 0.2 were used.

### Gene module enrichment analysis using Monocle

UT/IMQ-induced mouse *Pdgfra*-lineage cells scRNA-seq data and normal/psoriatic human skin fibroblasts extracted from the online database^25^ (GSE150672) were pre-clustered and labeled with Seurat according to the countermark genes, and were then converted as input for Monocle 3^63^. To find genes that vary in datasets, the graph_test function was performed with default parameters (*q* < 0.05). Co-regulated genes were grouped into gene modules using the find_gene_modules function with default parameters (resolution = 1e-2). All genes in top-ranked gene modules were output for Gene Ontology analysis using Metascape^64^ (version 3.5).

### Spatial transcriptomics

Fresh skin biopsies from healthy donors and patients with psoriasis were embedded in optical cutting tissue (OCT) compound and snap-frozen on dry ice. Skin sections (10 μm thick) were prepared using a cryostat microtome and mounted onto Visium slides (Visium Spatial Tissue Optimization Slide & Reagent kit, 10x Genomics). After hematoxylin and eosin (H&E) staining, bright-field images were obtained. Optimized permeabilization (for 24 min) and tissue removal were conducted on the Visium slides. After reverse transcription, the barcoded cDNA was enzymatically released and collected. The cDNA libraries were then sequenced on an Illumina NextSeq platform. The data were processed with the SpaceRanger (10x Genomics, version 1.1.0) and mapped to the GRCh38-2020-A genome. Results were visualized using the Seurat package (version 3.1.5).

### Flow cytometry

Single-cell suspensions were pelleted and resuspended in PBS with 2% FBS containing fluorophore-conjugated antibodies. Cells were initially stained with antibodies targeting cell surface proteins and with Live/dead Fixable Violet Dead Cell Stain Kit (Thermo Scientific, cat: L34964) for 30 min on ice and washed with PBS containing 2% FBS. For intracellular target staining, cells were then fixed and permeabilized using a Cytofix/Cytoperm kit (BD Biosciences, cat: 554714). For intracellular cytokine staining, single-cell suspensions were plated at 4 × 10^6^ cells per well in a 96-well round bottom plate, resuspended in 1× cell stimulation cocktail (plus protein transport inhibitors, eBioscience, cat: 00-4975-03) and incubated at 37 °C for 4 h before antibody staining. Samples were run on a BD Fortessa and analyzed using FlowJo software (Treestar, version 10.6.2).

The following antibodies were used for mouse flow cytometry: Alexa Fluor® 700 anti-mouse CD45 [Clone: 30-F11] (BioLegend, cat: 103128), PE CF594 anti-mouse Cd3e [Clone: 145 2C11] (BD, cat: 562286), PE-Cyanine5 anti-mouse TCR gamma/delta [Clone: GL-3] (Invitrogen, cat: 15-5711-81), PE anti-mouse IL-17A [Clone: TC11-18H10.1] (BioLegend, cat: 506904).

### Real-time quantitative PCR

Mouse skin specimens were snap-frozen in liquid nitrogen and pulverized. Total RNA was isolated using TRIzol Reagent (Invitrogen, cat: 15596026), quantified with a NanoDrop spectrophotometer, and reverse-transcribed into cDNA using an M-MLV First-Strand Synthesis Kit (Invitrogen, cat: C28025-021) with oligo(dT) primers. qPCR was conducted with the Hieff qPCR SYBR Green Master Mix (Yeasen Biotech, cat: 11202ES03) in a ViiA 7 Real-Time PCR system (Applied Biosystems). The relative expression of target genes was confirmed using the quantity of target gene/quantity of GAPDH. The following primers were used:

*Gapdh* forward: TCAATGAAGGGGTCGTTGAT,

*Gapdh* reverse: CGTCCCGTAGACAAAATGGT;

*Coch* forward: TTACCCCCTCGGAAACCTAC,

*Coch* reverse: ACACGTGGGTCTCGTTCAA;

*Tnc* forward: CACAACCCGTGAGTACCAGC,

*Tnc* reverse: AGAGGGTATGCTATAAGCCAGAA;

*Pdgfra* forward: AACGGAGGAGCTGCGGGGAA;

*Pdgfra* reverse: CCCATAGCTCCTGAGACCTTCTCCT;

*Itga7* forward: CTGCTGTGGAAGCTGGGATTC,

*Itga7* reverse: CTCCTCCTTGAACTGCTGTCG;

*Itga8* forward: TGGCTGGGATTCCAAGAGGA,

*Itga8* reverse: GTGCCCCGACCAATATGTCA;

*Itga9* forward: CACAACCCGTGAGTACCAGC,

*Itga9* reverse: GGTCTGCTTCGTAGTAGATGTTC;

*Itgav* forward: CCGTGGACTTCTTCGAGCC;

*Itgav* reverse: CTGTTGAATCAAACTCAATGGGC;

*Itgb1* forward: ATGCCAAATCTTGCGGAGAAT,

*Itgb1* reverse: TTTGCTGCGATTGGTGACATT;

*Itgb3* forward: ACGAGACCATCCTGTGTGAGCT;

*Itgb3* reverse: GCAAGTTGTCCTGGTGACTCGA.

*Itgb6* forward: CAACTATCGGCCAACTCATTGA;

*Itgb6* reverse: GCAGTTCTTCATAAGCGGAGAT.

### Western blotting

Mouse skin specimens and cultured cells were lysed in radioimmunoprecipitation buffer (Beyotime Biotechnology, cat: P0013B) supplemented with protease (ApexBio Technology, cat: K1007) and phosphatase inhibitor cocktails (ApexBio Technology, cat: K1015). Proteins were then separated by 10% SDS-PAGE gel and transferred onto the polyvinylidene difluoride membrane (Millipore®). Anti-Cochlin (Beyotime, cat: AF6522, 1:1000), anti-Tenascin-C (Cell Signaling Technology, cat: 12221S, 1:1000), anti-βIII tubulin (Abcam, cat: ab78078, 1:1000), anti-Phospho-ERK1/2 (Thr202/Tyr204; Cell Signaling Technology, cat: 9101S, 1:1000), and anti-GAPDH (Proteintech, cat: 60004-1-Ig, 1:50000) primary antibodies were used. The signals were detected with an enhanced ECL chemiluminescent substrate kit (Yeasen Biotechnology, cat: 36222) and imaged with GE Amersham Imager 600 (GE Healthcare).

### Histology and immunohistochemistry

Skin specimens were fixed in 4% paraformaldehyde and embedded in paraffin. For immunohistochemical staining, the sections (6 μm) were deparaffinized and washed in PBS. Antigen retrieval was performed by heating the sections in 10 mM sodium citrate buffer (pH = 6.0). The sections were washed in PBS after cooling, incubated in 3% hydrogen peroxide for 10 min at RT, and then washed again in PBS. The sections were blocked in blocking buffer (5% normal goat serum, 3% BSA, and 0.2% Triton X-100 in PBS) for 1 h at RT and stained in blocking buffer containing primary antibodies (anti-CD3, Servicebio Technology, cat: GB111337, 1: 1000; anti-CD45, Servicebio Technology, cat: GB113886, 1: 500) overnight at 4 °C. On the following day, the sections were warmed to RT for 1 h, washed three times in PBS, and stained with an HRP-polymer complex for 20 min, followed by incubation with a secondary antibody for 20 min. The sections were washed three times in PBS, developed with a DAB reagent (Peroxidase Substrate Kit, ZSGB-BIO, cat: ZLI-9018), and counterstained with hematoxylin. The sections were washed with tap water and then subsequent washes of increasing ethanol concentration for dehydration. Once mounted and air-dried, the slices were viewed under an Olympus BX53 microscope. To measure epidermal hyperplasia (acanthosis), images were taken after H&E staining, then the epidermis was outlined and measured with the lasso tool in Adobe Photoshop 2020. Pixels of relative areas of the epidermis were automatically calculated and converted into μm^2^ with the following formula: area (μm^2^) = 1.01^2^ × pixels.

### Immunofluorescence

Cryosections (10 μm) of mouse skin were cut using a cryostat (Leica, CM1950) and fixed in ice-cold acetone for 15 min. Human and mouse paraffin sections (6 μm) were dewaxed and antigen unmasked. The slices were blocked with blocking buffer(5% normal goat serum, 3% BSA, and 0.2% Triton X-100 in PBS) for 1 h and stained with anti-Cochlin (Beyotime Biotechnology, cat: AF6522, 1:100), anti-Tenascin C (Abcam, cat: ab10893, 1:100), anti-Ki67 (Servicebio Technology, cat: GB121141, 1:500) and anti-β-III tubulin (Abcam, cat: ab78078, 1:1000; ab18207, 1:100) primary antibodies overnight at 4 °C. Afterward, the slices were rinsed three times in PBS and stained with relative secondary antibodies (all from Invitrogen) for 1 h at RT. Nuclei were stained with DAPI (BD Biosciences, cat: 564907) at RT for 5 min. After being washed in PBS, the slices were mounted (Beyotime Biotechnology, cat: P0126) and visualized under a confocal microscopy system (Leica, TSC SP8).

### Imaging of bioluminescent reporter mice

Mice induced by IMQ for indicated days were imaged with an IVIS® Spectrum in vivo imaging system (PerkinElmer). Photon emission was detected with acquisition times ranging from 5 s to 3 min. The images were analyzed with Living Image software (PerkinElmer) by obtaining the average radiance per second per cm^2^ of specified regions of interest.

### Whole-mount immunofluorescence

Whole-mount samples were prepared according to *Rapiclear*® 1.52 Solution protocol. Briefly, euthanized C57BL/6 mice were given cardiac perfusion with PBS. Skin samples were then isolated and fixed with a 4% paraformaldehyde solution. After 3 times PBS wash, samples were treated with 2% PBST (2% Triton X-100, 0.05% sodium azide in PBS) for 2 days at RT for permeabilization. Thereafter, samples were washed with PBS and kept in blocking buffer on an orbital shaker for 2 days at 4 °C, then stained with anti-βIII tubulin (Abcam, cat: ab78078, 1:1000) and anti-CD3e (Proteintech, cat: 17617-1-AP, 1:100) for 2 days at 4 °C. After incubation, samples were washed with washing buffer 3 times in RT and then overnight at 4 °C. Afterward, samples were then incubated with a secondary antibody (Invitrogen) for 2 days at 4 °C and washed with washing buffer 3 times in RT then overnight at 4 °C. After 3 times PBS wash, samples were stained with DAPI at RT for 1.5hrs. Then, after PBS wash, samples were cleared with Rapiclear® reagent (SunJin Lab, cat: RC152001) and mounted in iSpacer (SunJin Lab, cat: IS007) microchamber. For skin samples from bioluminescent reporter mice, antibody incubation and related wash steps are omitted. Images were acquired using a confocal microscopy system (Leica, TSC SP8) with HC PL APO CS2 40×/1.30 OIL objective, with a scan speed of 200 Hz and a step size of 1.0 μm. The X/Y acquiring format was set as 1024*1024, and Z-stack size was determined by specimen thickness respectively.

### Mouse DRG neuron isolation and co-culture assay

DRG neurons were dissociated from DRGs of 6-week-old male C57BL/6 mice. Euthanized mice were given cardiac perfusion with PBS. DRGs were then dissociated and placed into an L-15 medium (Invitrogen, cat: 11415064) on ice for later digestion in DMEM containing 0.1 mg/ml DNase I (Sigma-Aldrich, cat: DN25), 0.4 mg/ml trypsin type I (Sigma-Aldrich, cat: T8003), and 1 mg/ml collagenase type IA (Sigma-Aldrich, cat: number C9891) at 37 °C for 35 min. Single-cell suspensions were plated on a glass-bottom cell culture dish (Nest Biotechnology, cat: 801001) or microfluidic chambers (Xona Microfluidics, cat: XC150) pre-seeded with monolayer NC/*Tnc*^OE^ NIH-3T3 cells. After 3 h, the medium was replaced with a neurobasal medium (Gibco, cat: 21103049,) containing B-27 supplement (Gibco, cat: 17504044), 2 mM l-glutamine (Gibco, cat: 25030081), 10% fetal bovine serum (Gibco, cat: 10099141C) and 1% antibiotic‒antimycotic (Gibco, cat: 15240062,). After 48–72 h of co-culture or stimulation with recombinant TNC (R&D systems, cat: 3358-TC), ERK agonist butylhydroquinone (TBHQ, MCE, cat:HY-100489) or ERK inhibitor AZD6244 (Slleck, cat:S1008), immunofluorescent staining assays were performed. Briefly, neurons were washed with PBS twice and fixed with 4% paraformaldehyde for 15 min, followed by blocking with 3% BSA for 1 h at RT. Cells were then incubated with anti-βIII tubulin (Abcam, cat: ab78078, 1:1000) at 4 °C overnight and labeled with secondary antibodies (Invitrogen) for 1 h at RT. DAPI incubation at RT for 5 min was used for nuclei staining. After being washed in PBS, samples were mounted with a fluorescent mounting medium. Images were acquired using a confocal microscopy system (Leica, TSC SP8), and fields were randomly taken and calculated. Average neurite areas were quantified by Image J (version 1.54b) and normalized to cell numbers. Axon lengths were calculated in Imaris (Bitplane, version 9.0.1).

### Quantification and statistical analysis

The data were analyzed using GraphPad Prism (version 8.0.2) and are presented as the mean ± SEM. A Student’s *t*-test was used to compare two conditions, and an analysis of variance (ANOVA) was used for multiple comparisons. A simple linear regression model was used to analyze the correlation between TNC expression and βIII tubulin expression. The probability values of <0.05 were considered statistically significant.

### Reporting summary

Further information on research design is available in the Nature Portfolio Reporting Summary linked to this article.

## Supplementary information

Supplementary information

Reporting Summary

PeerReviewFile

## Data Availability

The high-throughput sequencing data related to this study have been deposited in the GEO database^65^ under accession code GSE205020, GSE205790, GSE150672, and GSE131230. Source data are provided with this paper.

## Source data

Source Data
