# Supplementary material for: Tenascin C+ papillary fibroblasts facilitate neuro-immune interaction in a mouse model of psoriasis
Source: Nat Commun. 2023 Apr 10;14:2004. doi: 10.1038/s41467-023-37798-x (PMC10086024; doi:10.1038/s41467-023-37798-x)
Supplement: Supplementary file 1 — Supplementary information [file 41467_2023_37798_MOESM1_ESM.pdf]

Supplementary information

Tenascin C+ Papillary Fibroblasts Facilitate Neuro-immune Interaction in a Mouse Model of Psoriasis

Xiaojie CAI[1,*], Maoying HAN[2,*], Fangzhou LOU[1], Yang SUN[1], Qianqian YIN[3], Libo SUN[1], Zhikai WANG[3], Xiangxiao LI[1], Hong ZHOU[1], Zhenyao XU[1], Hong WANG[3], Siyu DENG[1], Xichen ZHENG[1], Taiyu ZHANG[1], Qun LI[4], Bin ZHOU[2,#], and Honglin WANG[1,5,#]

[1]Precision Research Center for Refractory Diseases, Shanghai General Hospital, Shanghai Jiao Tong University School of Medicine, Shanghai 201620, China

[2]State Key Laboratory of Cell Biology, CAS Center for Excellence in Molecular Cell Science, Shanghai Institute of Biochemistry and Cell Biology, Chinese Academy of Sciences, University of Chinese Academy of Sciences, Shanghai 200031, China

[3]Shanghai Institute of Immunology, Shanghai Jiao Tong University School of Medicine, Shanghai 200025, China

[4]The Department of Cardiovascular Medicine, State Key Laboratory of Medical Genomics, Ruijin Hospital, Shanghai Institute of Hypertension, Shanghai Jiao Tong University School of Medicine, Shanghai 200025, China

[5]Lead Contact

*These authors contributed equally.

#These authors jointly supervised this work:

Honglin WANG, Ph.D.
Precision Research Center for Refractory Diseases, Shanghai General Hospital, Shanghai Jiao Tong University School of Medicine, Shanghai 201620, China
E-mail: honglin.wang@sjtu.edu.cn

Bin ZHOU, Ph.D.
State Key Laboratory of Cell Biology, CAS Center for Excellence in Molecular Cell Science, Shanghai Institute of Biochemistry and Cell Biology, Chinese Academy of Sciences, University of Chinese Academy of Sciences, Shanghai 200031, China
E-mail: zhoubin@sibs.ac.cn

Supplementary Figure 1

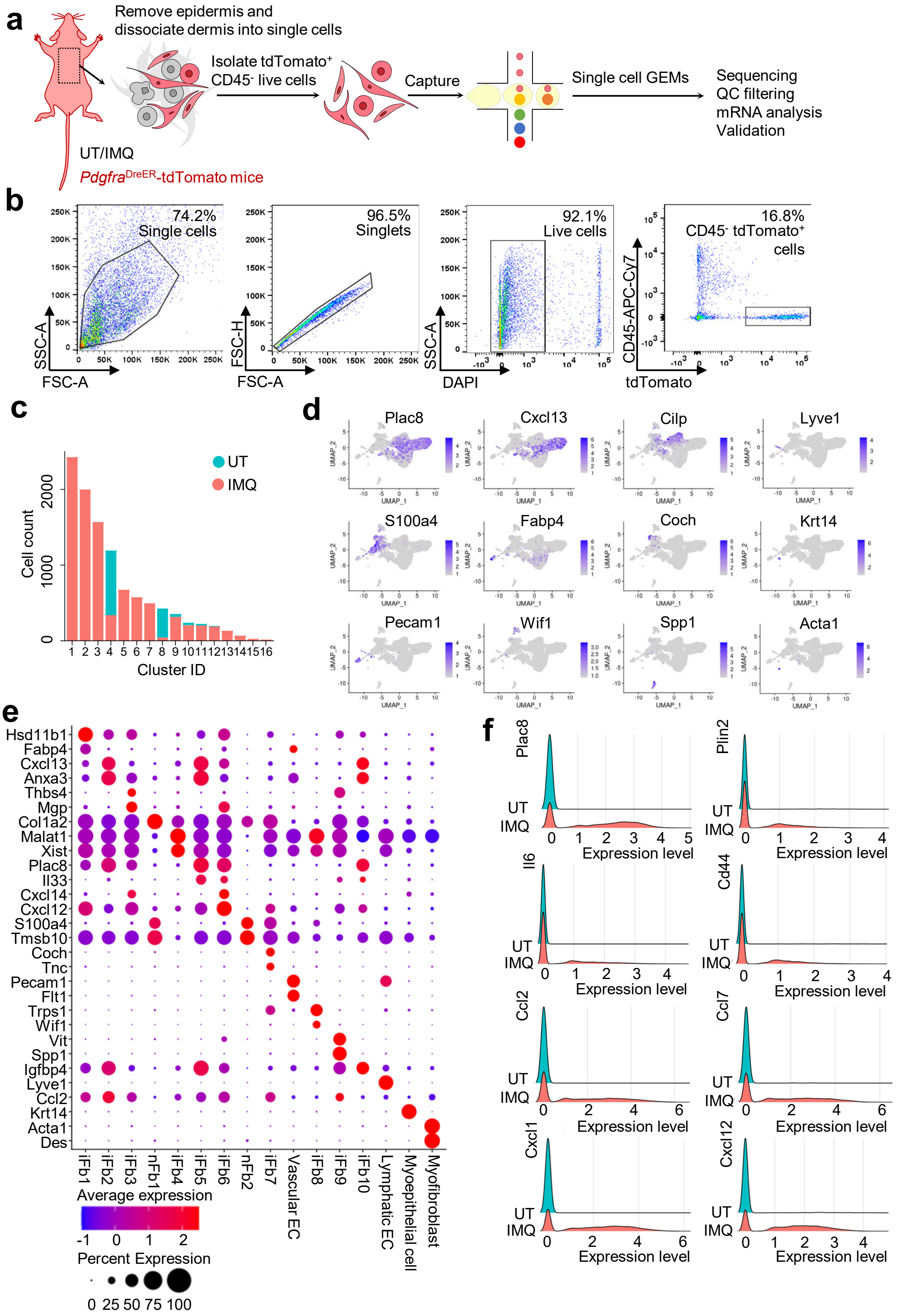

Supplementary Fig. 1: ScRNA-seq shows lineage skews and pro-inflammatory traits of dermal fibroblasts upon IMQ challenge.

a, ScRNA-seq workflow of *Pdgfra*-lineage cells from untreated (UT) and imiquimod (IMQ)-induced skin of *Pdgfra*^{DreER}-tdTomato mice (n = 1 per group).

b, Fluorescence-activated cell sorting gating strategy for CD45^{-}tdTomato^{+} lineage cells in (**a**).

c, Quantification of cell source among clusters (Of the total, 1340 UT cells and 9312 IMQ cells were included in statistics).

d, e, Feature plot (**d**) and dot plot (**e**) of signature genes among clusters.

f, Ridge plot of pro-adipogenic, pro-inflammatory and chemotactic gene expression level in cells from UT or IMQ dataset. Source data are provided as a Source Data file.

Supplementary Figure 2

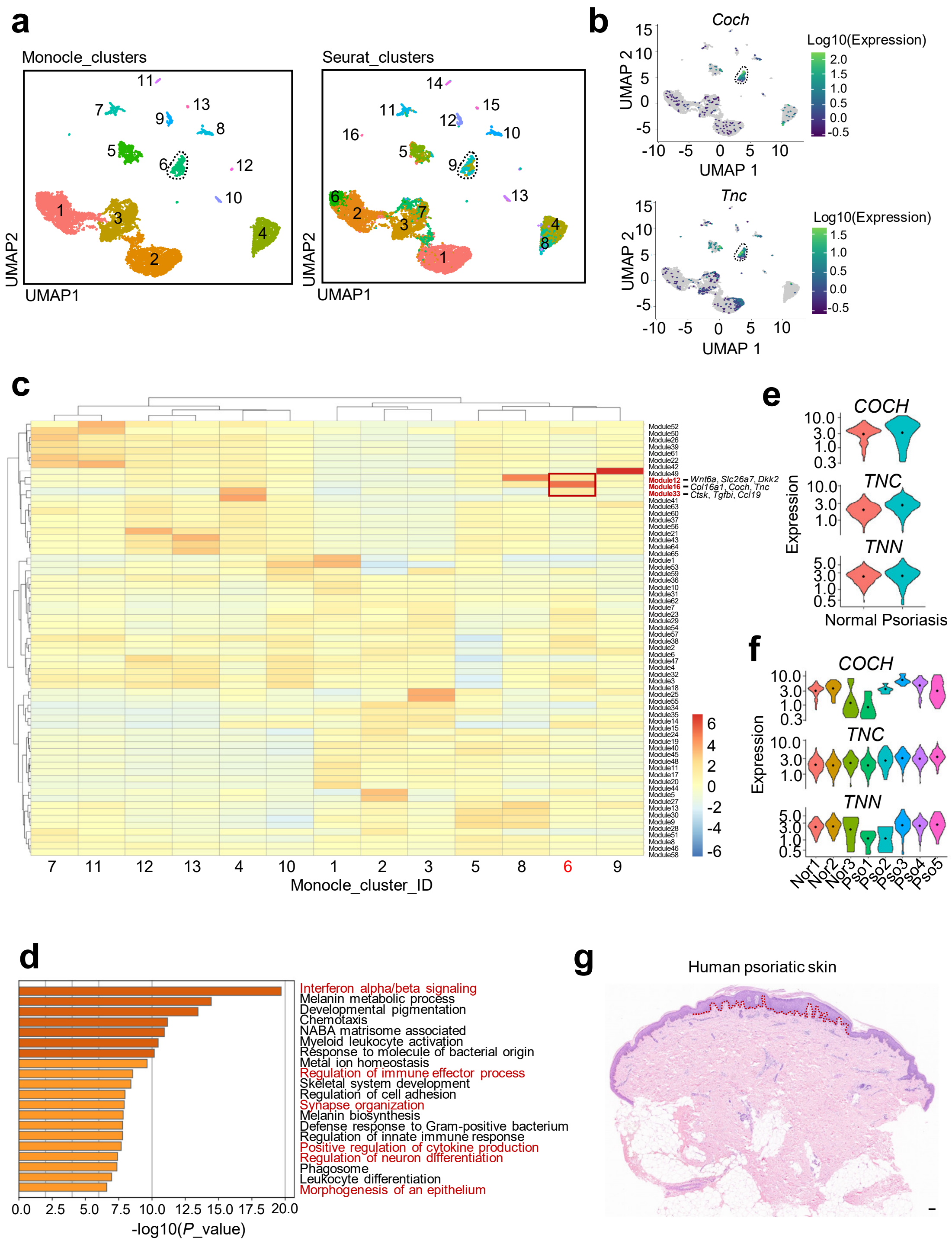

Supplementary Fig. 2: *Coch (COCH)*$^+$*Tnc (TNC)*$^+$ fibroblasts show neuron-regulatory and inflammation-responsive gene signatures in psoriatic skin.

a, Comparison of unbiased clustering of *Pdgfra*-lineage cells between Monocle and Seurat. *Coch*$^+$*Tnc*$^+$ fibroblast cluster is indicated by the dotted line.

b, Feature plots of *Coch* and *Tnc* by Monocle. *Coch*$^+$*Tnc*$^+$ fibroblast cluster is indicated by the dotted line.

c, Enriched gene module heatmap grouped by monocle clusters. The most enriched gene modules of *Coch*$^+$*Tnc*$^+$ fibroblasts are indicated by the red rectangle.

d-f, ScRNA-seq analysis of normal human skin versus psoriatic lesion. Top-ranked GO pathways of the enriched genes in *COCH*$^+$*TNC*$^+$*TNN*$^+$ dermal fibroblasts by Metascape (version 3.5, **d**). Violin plots of *COCH*, *TNC,* and *TNN* expression in normal (n = 3) or psoriatic skin (n = 5) fibroblasts in general (**e**) and individuals (**f**).

g, Representative H&E image of a human psoriatic skin biopsy (n = 5). The lesional area is indicated by the red dot line. Scale bar, 100 µm. Source data are provided as a Source Data file.

67 # Supplementary Figure 3

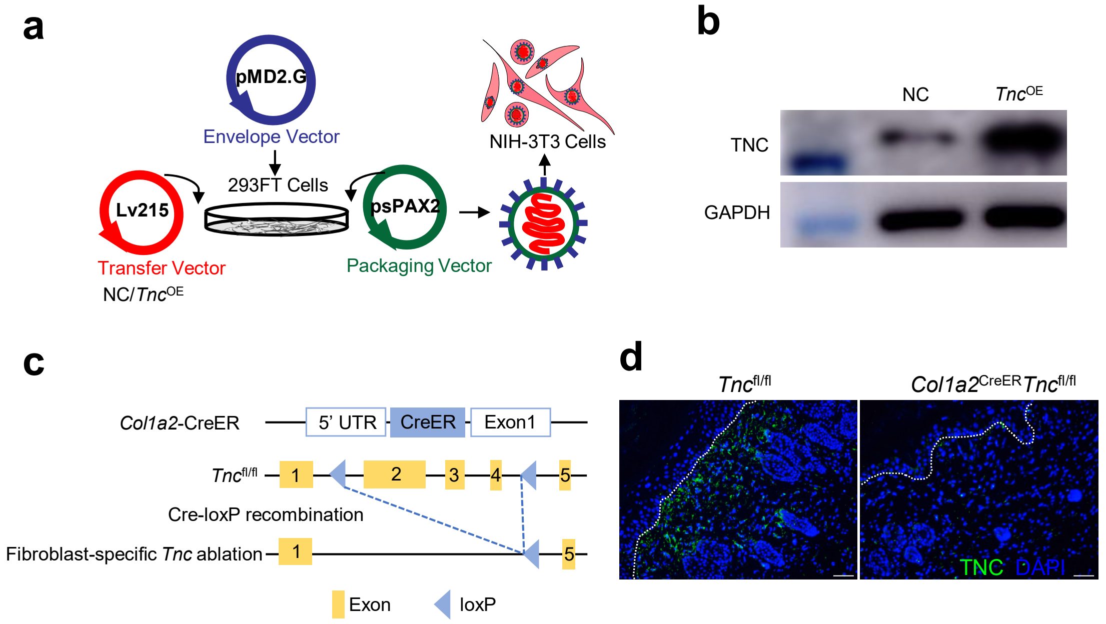

68

69

Supplementary Fig. 3: Construction strategies for the NC/Tnc$^{\mathbf{OE}}$ NIH-3T3 cell lines and *Col1a2*$^{\mathbf{CreER}}$*Tnc*$^{\mathbf{fl/fl}}$ mouse strain.

a, Construction strategy of NC/*Tnc*^{OE} NIH-3T3 cells by Lv215 lentivirus infection. NC, negative control. *Tnc*^{OE}, Tnc-overexpressing.

b, Immunoblot of TNC expression in NC and *Tnc*^{OE} NIH-3T3 cells.

c, Fibroblast-specific *Tnc* ablation strategy of *Col1a2*^{CreER}*Tnc*$^{\mathrm{fl/fl}}$ mice.

d, Representative immunofluorescent images of TNC expression in the skin of IMQ-induced *Tnc*$^{\mathrm{fl/fl}}$ or *Col1a2*^{CreER}*Tnc*$^{\mathrm{fl/fl}}$ mice (n = 3 mouse skin samples per group). Scale bar, 100 μm.

Data are representatives of two independent experiments.

Supplementary Figure 4

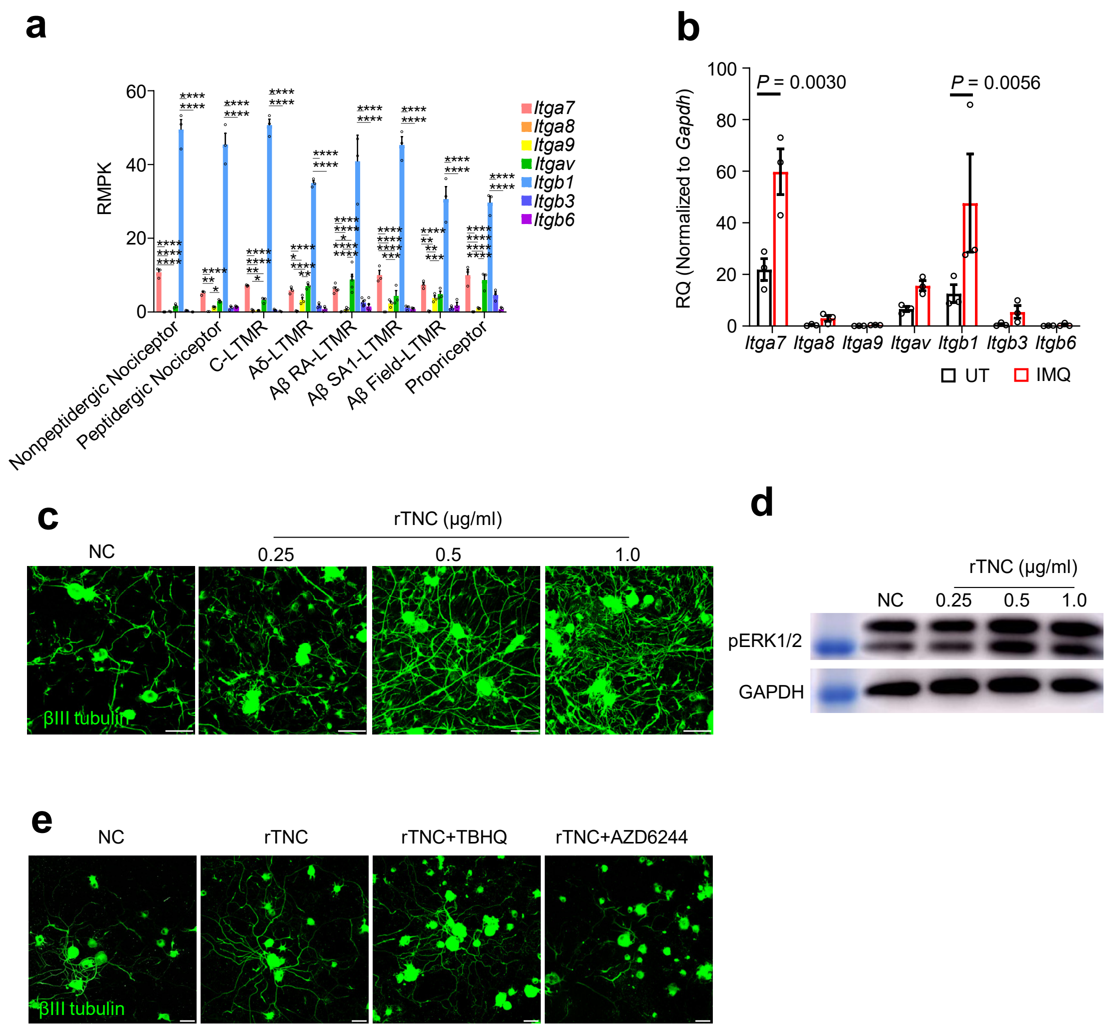

Supplementary Fig. 4: TNC promotes axonogenesis in an ERK signaling-dependent manner.

a, Quantification of integrin subunit expression on different dorsal root ganglion (DRG) neuron subtypes (n = 5 samples for Aβ RA-LTMR group, and n = 3 samples for other groups). Peptidergic Nociceptor, *$P = 0.0437$, **$P = 0.0022$; C-LTMR, *$P = 0.0424$, **$P = 0.0019$; Aδ-LTMR, *$P = 0.0285$, **$P = 0.0027$; Aβ RA-LTMR, *$P = 0.0149$; Aβ SA1-LTMR, ***$P = 0.0006$; Aβ Field-LTMR, **$P = 0.0054$ (*Itga7* vs. *Itga9*), **$P = 0.0060$ (*Itga8* vs. *Itga9*), ***$P = 0.0002$; For all groups, ****$P < 0.0001$.

b, qPCR analysis of integrin subunit expression of isolated DRG neurons from Untreated (UT) or Imiquimod-induced (IMQ) mice (n = 3 mice per group).

c, Representative immunofluorescent images of neurite outgrowth of DRG neurons treated with different doses of recombinant TNC (n = 3 cultures per group). Scale bar, 50 μm.

d, Immunoblot of ERK1/2 phosphorylation of DRG neurons treated with different doses of recombinant TNC.

e, Representative immunofluorescent images of neurite outgrowth of DRG neurons treated with recombinant TNC (0.5 μg/mL), ERK agonist butylhydroquinone (TBHQ, 1 μM) and ERK inhibitor AZD6244 (1 μM, n = 3 cultures per group). Scale bar, 50 μm. Data are representatives of two independent experiments and presented as the mean ± SEM. The P values were calculated by two-way ANOVA and Holm-Šídák test (**a**) or two-tailed unpaired Student's *t*-test (**b**). Source data are provided as a Source Data file.

Supplementary Figure 5

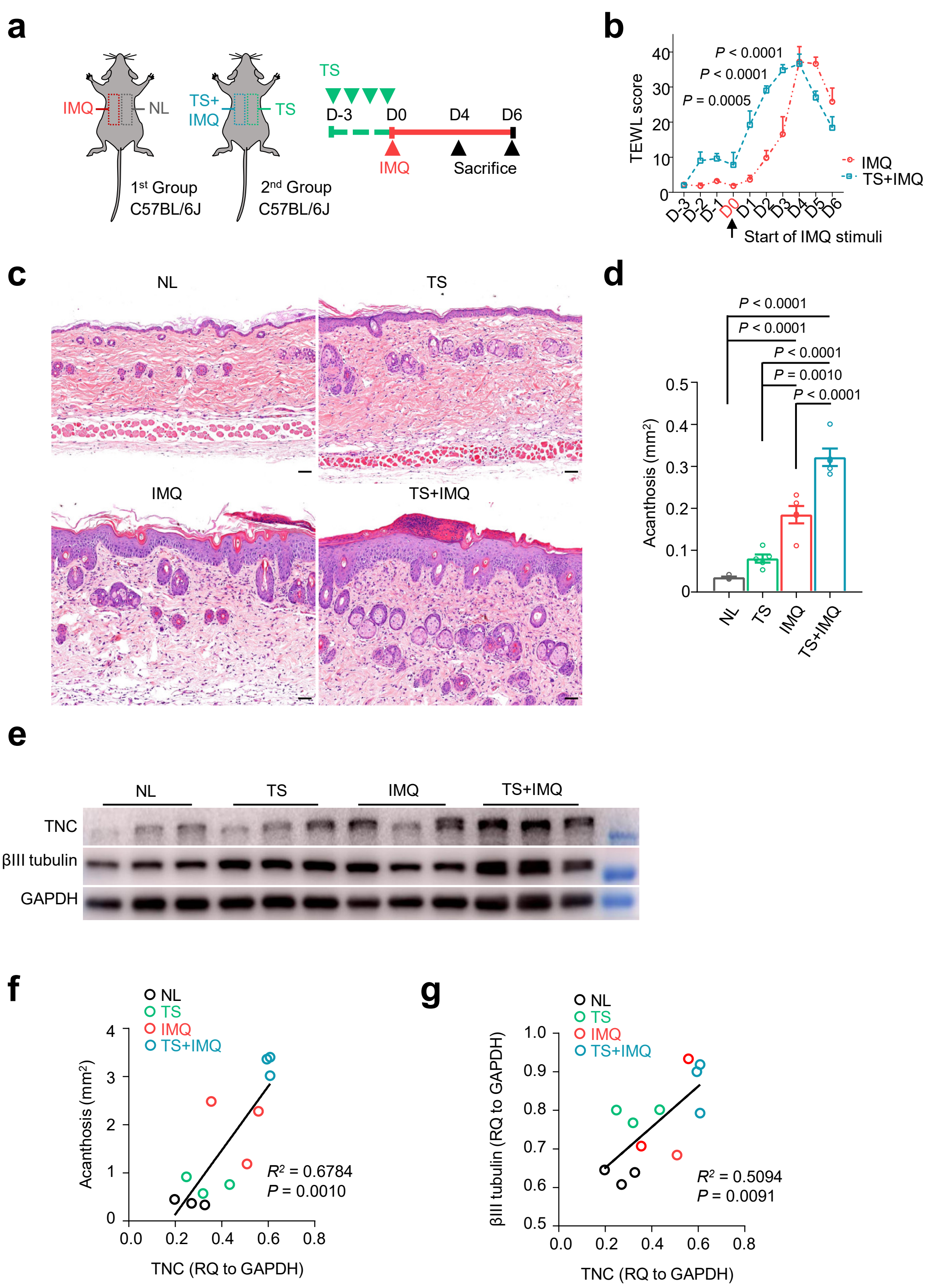

Supplementary Fig. 5: TNC and βIII tubulin expression are positively correlated upon injury and IMQ stimuli.

a, Schematic diagram of tape stripping (TS) pre-treated IMQ mouse model. No pre-TS in the 1st group; pre-TS 5 times daily for consecutive 4 days in the 2nd group. All mice were administrated with IMQ on half-back skin from D0 and were sacrificed on indicated days. NL, with no TS or IMQ treatment; IMQ, with only IMQ treatment; TS, with only TS treatment; TS+IMQ, with both TS and IMQ treatment.

b, Trans-epidermal water loss (TEWL) scores of TS or IMQ+TS mouse skin (n = 5 mice per group).

c, d, Representative H&E images (**c**) and quantification of acanthosis (**d**) of NL/TS/IMQ/TS+IMQ mouse skin on day 4 post IMQ administration (n= 5 mouse skin samples per group). Scale bar in (**c**), 50 μm.

e-g, Immunoblotting (**e**) and correlation analysis of TNC expression and acanthosis (**f**) or TNC and βIII tubulin expression (**g**) in NL/TS/IMQ/TS+IMQ mouse skin on day 4 post-IMQ administration (n= 3 mouse skin samples per group).

All data are representatives of two independent experiments. Data are presented as mean ± SEM in (**b, d**). The *P* values were calculated by two-way ANOVA and Holm-Šídák test (**b**), one-way ANOVA and Tukey's test (**d**), and Linear regression (**f, g**). Source data are provided as a Source Data file.

Supplementary Figure 6

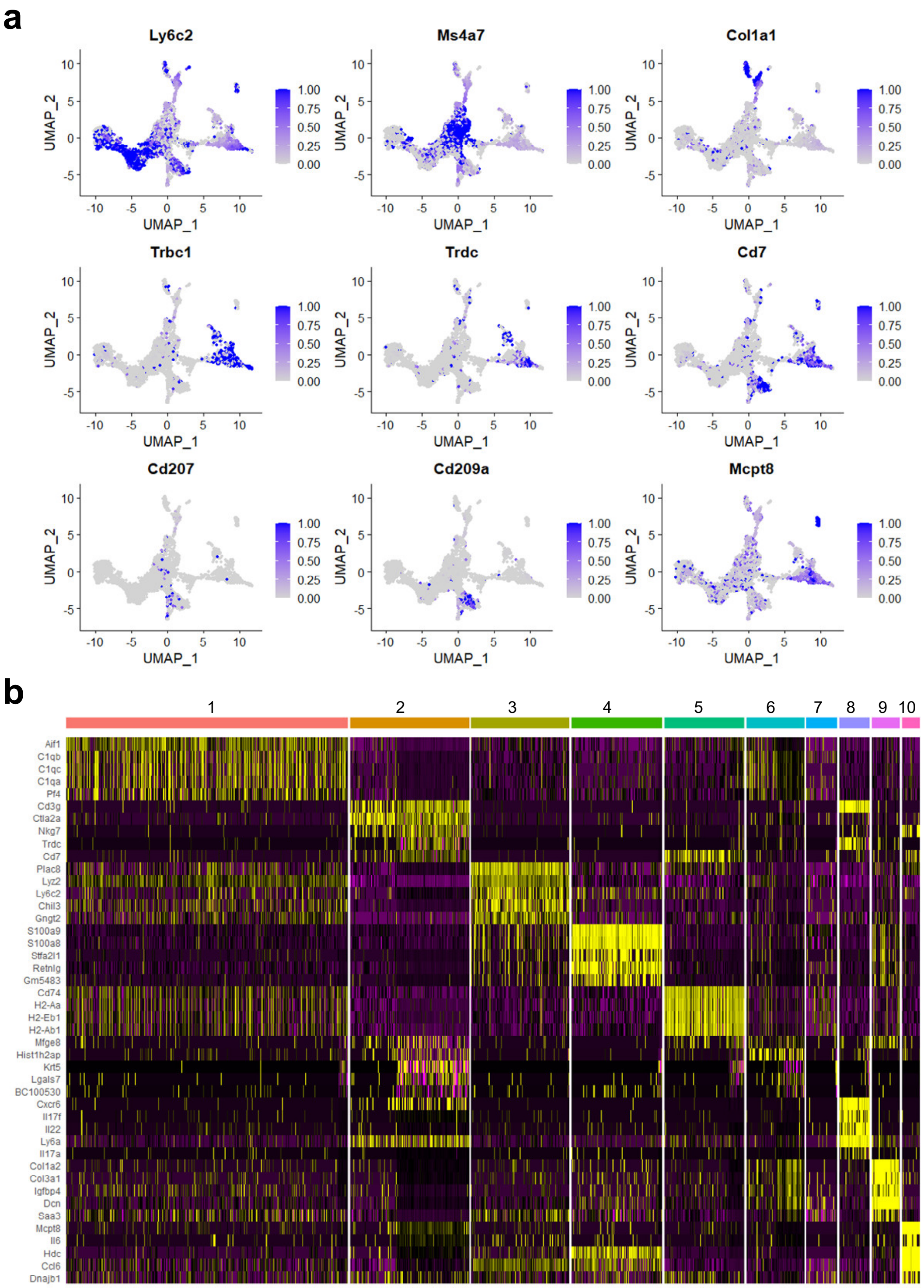

Supplementary Fig. 6: ScRNA-seq analysis of immune cells in IMQ-induced mouse skin.

a, b, Feature plots (**a**) and heatmap (**b**) of signature genes among clusters.

Supplementary Figure 7

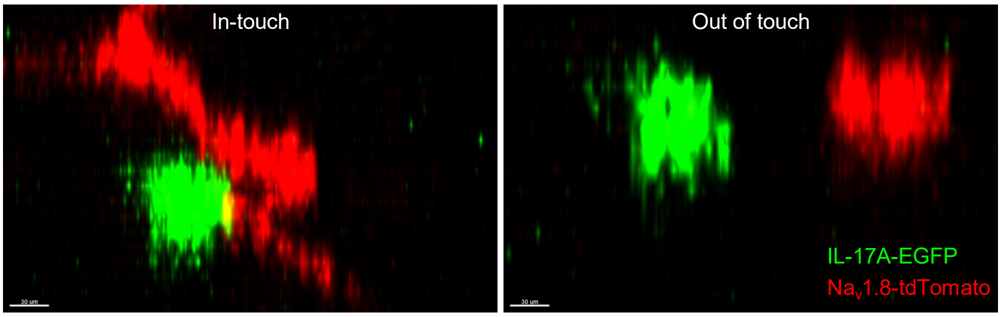

Supplementary Fig. 7: Pathogenic T cells in contact with cutaneous nerves in mouse psoriasiform lesions. Representative images of the IL-17A^{+} T cells that are in contact (left) or not in contact (right) with cutaneous nerves (over 300 EGFP^{+} T cells were observed during contact ratio quantification).

Supplementary Figure 8

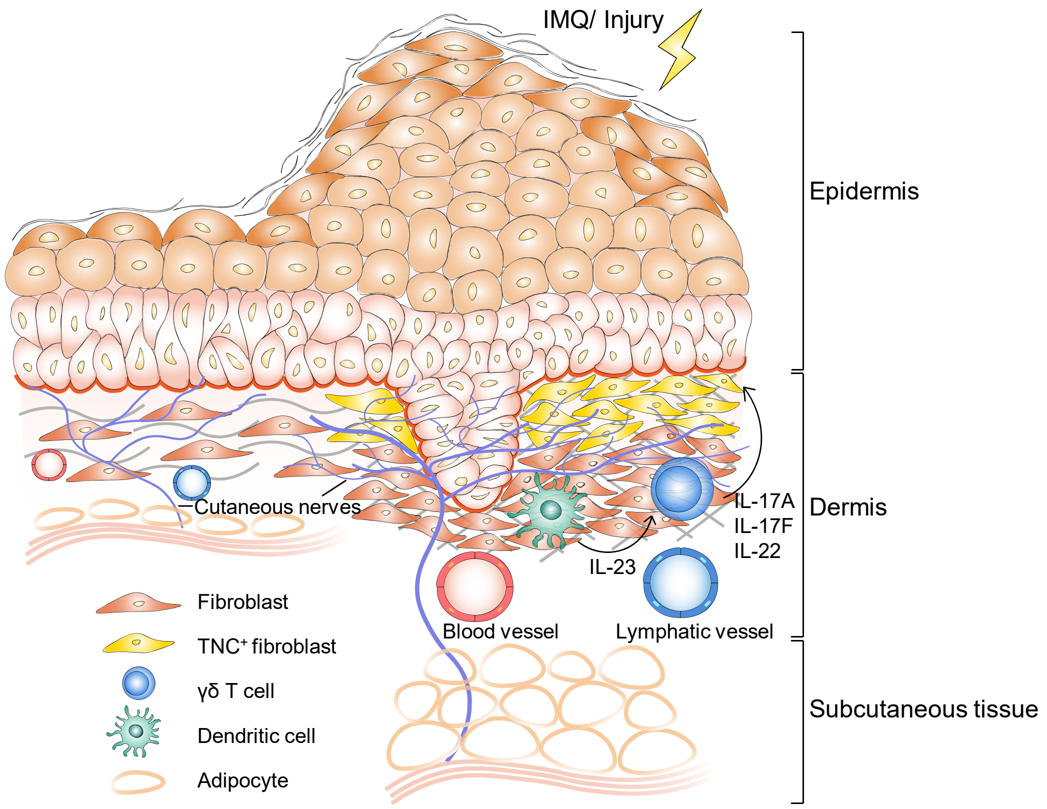

Supplementary Fig. 8: Graphical summary. A subset of TNC^+ fibroblasts emerged at the dermal-epidermal junction upon skin irritation and promoted inflammation through facilitating excessive axonogenesis and neuro-immune synapse formation.

Supplementary Figure 9

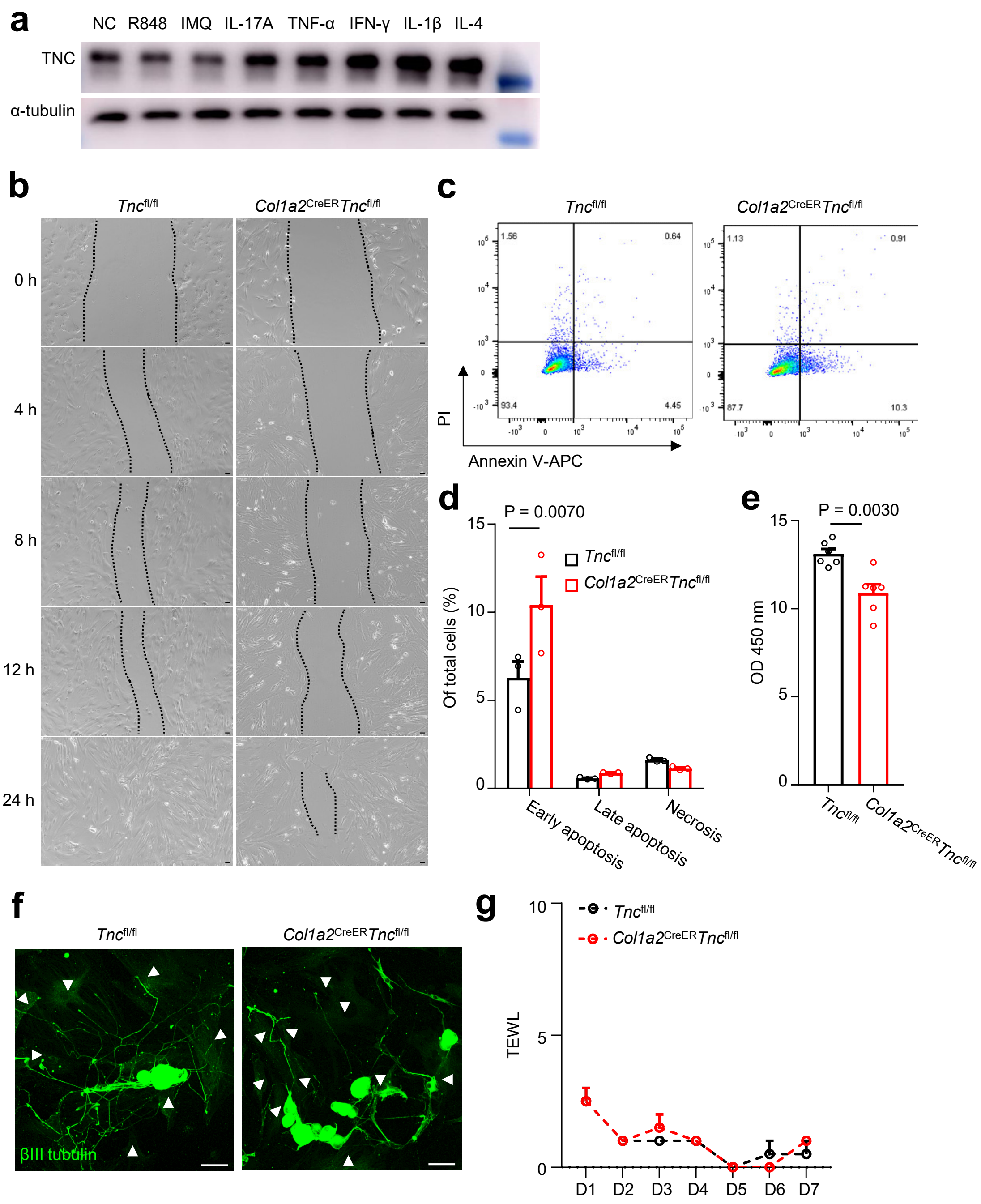

Supplementary Fig. 9: The effect of TNC ablation on primary mouse dermal fibroblasts. *(Related to the point-by-point response letter to the reviewers)*

a, Immunoblot of TNC expression in primary mouse dermal fibroblasts treated with imiquimod (IMQ, 2 μg/mL), resiquimod (R848, 1 μg/mL), recombinant IL-17A and several other inflammatory cytokines (10 ng/mL).

b, Scratch assay images of primary dermal fibroblasts from *Tnc*$^{\text{fl/fl}}$ or *Col1a2*^{CreER}*Tnc*$^{\text{fl/fl}}$ mice (n = 3). Scale bar, 10000 μm.

c, d, Flow cytometric analysis (**c**) and quantification (**d**) of primary dermal fibroblasts apoptosis from *Tnc*$^{\text{fl/fl}}$ or *Col1a2*^{CreER}*Tnc*$^{\text{fl/fl}}$ mice (n = 3).

e, CCK8 quantification of primary dermal fibroblast proliferation from *Tnc*$^{\text{fl/fl}}$ or *Col1a2*^{CreER}*Tnc*$^{\text{fl/fl}}$ mice (n = 6).

f, Representative immunofluorescent images of dorsal root ganglion (DRG) neurons co-cultured with primary dermal fibroblasts from *Tnc*$^{\text{fl/fl}}$ or *Col1a2*^{CreER}*Tnc*$^{\text{fl/fl}}$ mice (n = 3). Surrounding fibroblasts were labeled by white arrowheads. Scale bar, 50 μm**.**

g, Trans-epidermal water loss (TEWL) scores of *Tnc*$^{\text{fl/fl}}$ or *Col1a2*^{CreER}*Tnc*$^{\text{fl/fl}}$ mouse shaved back skin (n = 3).

All data are representatives of two independent experiments. Data are presented as mean ± SEM in (**d, e, g**). The *P* values were calculated by two-way ANOVA and Holm-Šídák test (**d, g**) or two-tailed unpaired Student's *t*-test (**e**).

Source data are provided as a Source Data file.
