# Supplementary material for: Tenascin C+ papillary fibroblasts facilitate neuro-immune interaction in a mouse model of psoriasis
Source: Nat Commun. 2023 Apr 10;14:2004. doi: 10.1038/s41467-023-37798-x (PMC10086024; doi:10.1038/s41467-023-37798-x)
Supplement: Supplementary file 2 — Reporting Summary [file 41467_2023_37798_MOESM2_ESM.pdf]

nature portfolio

Corresponding author(s): Honglin Wang

Last updated by author(s): Mar 2, 2023

Reporting Summary

Nature Portfolio wishes to improve the reproducibility of the work that we publish. This form provides structure for consistency and transparency in reporting. For further information on Nature Portfolio policies, see our Editorial Policies and the Editorial Policy Checklist.

Statistics

For all statistical analyses, confirm that the following items are present in the figure legend, table legend, main text, or Methods section.

n/a	Confirmed	
☐	☒	The exact sample size (*n*) for each experimental group/condition, given as a discrete number and unit of measurement
☐	☒	A statement on whether measurements were taken from distinct samples or whether the same sample was measured repeatedly
☐	☒	The statistical test(s) used AND whether they are one- or two-sided<br>*Only common tests should be described solely by name; describe more complex techniques in the Methods section.*
☐	☒	A description of all covariates tested
☐	☒	A description of any assumptions or corrections, such as tests of normality and adjustment for multiple comparisons
☐	☒	A full description of the statistical parameters including central tendency (e.g. means) or other basic estimates (e.g. regression coefficient) AND variation (e.g. standard deviation) or associated estimates of uncertainty (e.g. confidence intervals)
☐	☒	For null hypothesis testing, the test statistic (e.g. *F*, *t*, *r*) with confidence intervals, effect sizes, degrees of freedom and *P* value noted<br>*Give P values as exact values whenever suitable.*
☒	☐	For Bayesian analysis, information on the choice of priors and Markov chain Monte Carlo settings
☒	☐	For hierarchical and complex designs, identification of the appropriate level for tests and full reporting of outcomes
☒	☐	Estimates of effect sizes (e.g. Cohen's *d*, Pearson's *r*), indicating how they were calculated

Our web collection on statistics for biologists contains articles on many of the points above.

Software and code

Policy information about availability of computer code

Data collection	For single-cell RNA sequencing, the constructed libraries were sequenced with an Illumina HiSeq 4000 platform, and the trimmed data were processed using the CellRanger (version 3.0, 10x Genomics). For spatial transcriptomics, the cDNA libraries were sequenced on an Illumina NextSeq platform, and the data were processed with the SpaceRanger (version 1.1.0, 10x Genomics). The real-time quantitative PCR data was collected in a ViiA 7 Real-Time PCR system. Western blotting signals were imaged with GE Amersham Imager 600. Histological and immunohistochemistry images were captured by an Olympus BX53 microscope. Immunofluorescent and whole-mount images were captured by a Leica TSC SP8 confocal microscopy system. Luminescence images of reporter mice were taken with an PerkinElmer IVIS® Spectrum in vivo imaging system.
Data analysis	The single-cell RNA sequencing data were analyzed by R (version 3.6.3) using the Seurat package (version 3.1.2) and Monocle 3. The spatial transcriptomic data were processed with SpaceRanger (version 1.1.0) and visualized using the Seurat package (version 3.1.5). The Gene Ontology analysis were performed with Metascape (version 3.5). The immunofluorescent images were visualized by Leica LAZ X and Bitplane Imaris (version 9.0.1). Average neurite areas were quantified by Image J (version 1.54b). Axon lengths were calculated in Bitplane Imaris (version 9.0.1). The data were analyzed using GraphPad Prism (Version 8.0.2).

For manuscripts utilizing custom algorithms or software that are central to the research but not yet described in published literature, software must be made available to editors and reviewers. We strongly encourage code deposition in a community repository (e.g. GitHub). See the Nature Portfolio guidelines for submitting code & software for further information.

Data

Policy information about availability of data

All manuscripts must include a data availability statement. This statement should provide the following information, where applicable:

- Accession codes, unique identifiers, or web links for publicly available datasets
- A description of any restrictions on data availability
- For clinical datasets or third party data, please ensure that the statement adheres to our policy

The high-throughput sequencing data related to this study have been deposited in the GEO database under accession code GSE205020 [https://www.ncbi.nlm.nih.gov/geo/query/acc.cgi?acc=GSE205020], GSE205790 [https://www.ncbi.nlm.nih.gov/geo/query/acc.cgi?acc=GSE205790], GSE150672 [https://www.ncbi.nlm.nih.gov/geo/query/acc.cgi?acc=GSE150672], and GSE131230 [https://www.ncbi.nlm.nih.gov/geo/query/acc.cgi?acc=GSE131230]. Source data are provided with this paper.

Human research participants

Policy information about studies involving human research participants and Sex and Gender in Research.

Reporting on sex and gender	This information has not been collected.
Population characteristics	Treatment-naïve patients with psoriasis vulgaris (age 38-59) and control healthy donors who had surgeries(age 25-55) were recruited in this study for skin biopsy collection.
Recruitment	Psoriasis patients with 3-month washout period were randomly recruited. All patients have been diagnosed with moderate-to-severe psoriasis with body surface area ≥10% and PASI ≥12. Normal human skin specimens were taken from healthy donors who underwent plastic surgery.
Ethics oversight	Analysis studies involving human skin biopsies were reviewed and approved by the Ethics Committee of Shanghai General Hospital, China (No. 2018KY239).

Note that full information on the approval of the study protocol must also be provided in the manuscript.

Field-specific reporting

Please select the one below that is the best fit for your research. If you are not sure, read the appropriate sections before making your selection.

☒ Life sciences ☐ Behavioural & social sciences ☐ Ecological, evolutionary & environmental sciences

For a reference copy of the document with all sections, see nature.com/documents/nr-reporting-summary-flat.pdf

Life sciences study design

All studies must disclose on these points even when the disclosure is negative.

Sample size	Sample size (n ≥ 3) used for animal experiments was commonly accepted based on variance for the type of experiments and availability of germ-free mice with matched age and genotypes. Bstatistical criteria.
Data exclusions	No data were excluded except for single-cell RNA sequencing analysis: cells with fewer than 200 genes, more than 5,000 genes, more than 5% mitochondria content, and doublets were removed as recommended.
Replication	All experiments were performed with independent replicates as described in the figure legends.
Randomization	Mice with comparable age, weight and indicated genotypes were randomly selected from housing cages and then assigned in groups with no bias for further treatment. Specimens for immunohistochemical staining were randomized chosen and assayed together.
Blinding	Investigators were not blind to group allocation.

Reporting for specific materials, systems and methods

We require information from authors about some types of materials, experimental systems and methods used in many studies. Here, indicate whether each material, system or method listed is relevant to your study. If you are not sure if a list item applies to your research, read the appropriate section before selecting a response.

Materials & experimental systems

n/a	Involved in the study
	☒ Antibodies
	☒ Eukaryotic cell lines
☒	Palaeontology and archaeology
	☒ Animals and other organisms
☒	Clinical data
☒	Dual use research of concern

Methods

n/a	Involved in the study
☒	ChIP-seq
	☒ Flow cytometry
☒	MRI-based neuroimaging

Antibodies

Antibodies used

Anti-Cochlin (Beyotime, cat: AF6522, WB-1:1000/IF-1:100)
Anti-Tenascin-C (Cell Signaling Technology, cat: 12221S, WB-1:1000)
Anti-Tenascin C (Abcam, cat: ab108930, WB-1:1000/IF-1:100)
Anti-βIII tubulin (Abcam, cat: ab78078, WB-1:1000/IF-1:1000)
Anti-βIII tubulin (Abcam, cat: ab18207, WB-1:1000/IF-1:100)
Anti-GAPDH (Proteintech, cat: 60004-1-Ig, WB-1:50000)
Anti-CD3 (Servicebio Technology, cat: GB111337, IHC-1:1000)
Anti-CD45 (Servicebio Technology, cat: GB113886, IHC-1:500)
Anti-Ki67 (Servicebio Technology, cat: GB121141, IF-1:500)
Anti-CD3e (Proteintech, cat: 17617-1-AP,IF-1:100)
Anti-Phospho-ERK1/2 (Thr202/Tyr204; Cell Signaling Technology, cat: 9101S, WB-1:1000)
Alexa Fluor® 700 anti-mouse CD45 [Clone: 30-F11] (BioLegend, cat: 103128, FCM-1:200)
PE CF594 anti-mouse Cd3e [Clone: 145 2C11] (BD, cat: 562286, FCM-1:200)
PE-Cyanine5 anti-mouse TCR gamma/delta [Clone: GL-3] (Invitrogen, cat: 15-5711-81, FCM-1:200)
PE anti-mouse IL-17A [Clone: TC11-18H10.1] (BioLegend, cat: 506904, FCM-1:200)

Validation

Anti-Cochlin (Beyotime, cat: AF6522, https://www.beyotime.com/product/AF6522.htm)
Anti-Tenascin-C (Cell Signaling Technology, cat: 12221S, https://www.cellsignal.cn/products/primary-antibodies/tenascin-c-d16c4-rabbit-mab/12221)
Anti-Tenascin C (Abcam, cat: ab108930, https://www.abcam.cn/products/primary-antibodies/tenascin-c-antibody-epr4219-ab108930.html)
Anti-βIII tubulin (Abcam, cat: ab78078, https://www.abcam.cn/products/primary-antibodies/beta-iii-tubulin-antibody-2g10-neuronal-marker-ab78078.html)
Anti-βIII tubulin (Abcam, cat: ab18207, https://www.abcam.cn/products/primary-antibodies/beta-iii-tubulin-antibody-neuronal-marker-ab18207.html)
Anti-GAPDH (Proteintech, cat: 60004-1-Ig, https://www.ptgcn.com/products/GAPDH-Antibody-60004-1-Ig.htm)
Anti-CD3 (Servicebio Technology, cat: GB111337, https://www.servicebio.com/goodsdetail?id=3689)
Anti-CD45, Servicebio Technology, cat: GB113886, https://www.servicebio.com/goodsdetail?id=7318)
Anti-Ki67 (Servicebio Technology, cat: GB121141, https://www.servicebio.com/goodsdetail?id=2828)
Anti-CD3e (Proteintech, cat: 17617-1-AP, https://www.thermofisher.cn/cn/zh/antibody/product/CD3-Antibody-Polyclonal/17617-1-AP)
Anti-Phospho-ERK1/2 (Thr202/Tyr204; Cell Signaling Technology, cat: 9101S, https://www.cellsignal.cn/products/primary-antibodies/phospho-p44-42-mapk-erk1-2-thr202-tyr204-antibody/9101)
Alexa Fluor® 700 anti-mouse CD45 [Clone: 30-F11] (BioLegend, cat: 103128, https://www.biolegend.com/en-us/products/alexa-fluor-700-anti-mouse-cd45-antibody-3407?GroupID=BLG6833)
PE CF594 anti-mouse Cd3e [Clone: 145 2C11] (BD, cat: 562286, https://www.bdbiosciences.com/zh-cn/products/reagents/flow-cytometry-reagents/research-reagents/single-color-antibodies-ruo/pe-cf594-hamster-anti-mouse-cd3e.562286)
PE-Cyanine5 anti-mouse TCR gamma/delta [Clone: GL-3] (Invitrogen, cat: 15-5711-81, https://www.thermofisher.cn/cn/zh/antibody/product/TCR-gamma-delta-Antibody-clone-eBioGL3-GL-3-GL3-Monoclonal/15-5711-81)
PE anti-mouse IL-17A [Clone: TC11-18H10.1] (BioLegend, cat: 506904, https://www.biolegend.com/ja-jp/products/pe-anti-mouse-il-17a-antibody-1633?GroupID=GROUP24)

Eukaryotic cell lines

Policy information about cell lines and Sex and Gender in Research

Cell line source(s)

Human 293FT (Invitrogen, cat: R70007) and mouse NIH-3T3 (ATCC, cat: CRL-1658) was used.

Authentication

Authentication for human 293FT was provide on Invitrogen website, https://www.thermofisher.cn/order/catalog/product/cn/zh/R70007; authentication for mouse NIH-3T3 was provided on ATCC website, https://www.atcc.org/products/crl-1658.

Mycoplasma contamination

All cells were tested negative for mycoplasma contamination with MycAwayTM Plus-Color ONE-Step Mycoplasma Detection Kit (Vazyme, cat: D101-01).

Commonly misidentified lines
(See ICLAC register)

No commonly misidentified cell lines were used.

Animals and other research organisms

Policy information about studies involving animals; ARRIVE guidelines recommended for reporting animal research, and Sex and Gender in Research

Laboratory animals	C57BL/6 mice, 6-8 weeks old, were purchased from Shanghai SLAC Laboratory Animal Co., Ltd. (Shanghai, China). Col1a2-CreER , Pdgfra-DreER, R26R-rox-tdTomato, R26R-tdTomato mouse line were previously described. EMBO J 39, e102675 (2020) ; J Clin Invest 127, 2968-2981 (2017); Nat Neurosci 13, 133-140 (2010); Circ Res 118, 254-265 (2016). Nav1.8-Cre mouse line60 was provided by Dr. Xiaoyang Cheng (Department of Anatomy, Histology, and Embryology, Shanghai Jiao Tong University School of Medicine, China). IL17-GFP mouse line (MGI: J:184819) was provided by Dr. Zhinan Yin (The First Affiliated Hospital, Biomedical Translational Research Institute and Guangdong Province Key Laboratory of Molecular Immunology and Antibody Engineering, Jinan University, Guangzhou, China). Tnc-DreER was generated by homologous recombination using CRISPR-Cas9 technology. In short, a cDNA encoding P2A-DreER-WPRE-polyA was inserted after coding region of Tnc gene before 3'UTR. Tncfl/fl mouse line was purchase from Cyagen Biosciences Inc (cat: CKOCMP-21923-Tnc-B6J-VA). PdgfraDreER-tdTomato and TncDreER-tdTomato mice were generated by crossing the PdgfraDreER or TncDreER with the R26R-rox-tdTomato reporter line, respectively. Nav1.8-tdTomato mice were generated by crossing the Nav1.8-Cre with the R26R-tdTomato reporter line, and were further crossed with the IL17-GFP line to obtain the Nav1.8tdTomatoIL17GFP dual reporter mice. Col1a2CreERTncfl/fl mice were generated by crossing the Col1a2-CreER with the Tncfl/fl mouse line. The mice were bred and maintained under specific pathogen-free (SPF) conditions. Mice were housed in cages with five mice per cage and kept on in a regular 12 hour:12 hour light: dark cycle. The temperature was 22±1 degree Celsius and humidity was 40-70%.
Wild animals	The study did not involved wild animals.
Reporting on sex	Male mice were used in the experiments if no otherwise noted. Age-matched and sex-matched (both male and female) Col1a2CreERTncfl/fl and Tncfl/fl mice were used to test the effect of Tnc conditional knock out in IMQ-induced psoriasiform skin inflammation.
Field-collected samples	The study did not involve samples collected from the field.
Ethics oversight	All of the experiments were approved by the National Institutes of Health Guide for the Care and Use of Laboratory Animals with the approval (SYXK- 2003-0026) of the Scientific Investigation Board of Shanghai Jiao Tong University School of Medicine in Shanghai, China.

Note that full information on the approval of the study protocol must also be provided in the manuscript.

Flow Cytometry

Plots

Confirm that:

- [x] The axis labels state the marker and fluorochrome used (e.g. CD4-FITC).
- [x] The axis scales are clearly visible. Include numbers along axes only for bottom left plot of group (a 'group' is an analysis of identical markers).
- [x] All plots are contour plots with outliers or pseudocolor plots.
- [x] A numerical value for number of cells or percentage (with statistics) is provided.

Methodology

Sample preparation	Single-cell suspensions were pelleted and resuspended in PBS with 2% FBS containing fluorophore-conjugated antibodies. Cells were initially stained with antibodies targeting cell surface proteins and with Live/dead Fixable Violet Dead Cell Stain Kit (Thermo Scientific, cat: L34964) for 30 min on ice and washed with PBS containing 2% FBS. For intracellular target staining, cells were then fixed and permeabilized using a Cytofix/Cytoperm kit (BD Biosciences, cat: 554714). For intracellular cytokine staining, single-cell suspensions were plated at 4×106 cells per well in a 96-well round bottom plate, resuspended in 1× cell stimulation cocktail (plus protein transport inhibitors, eBioscience, cat: 00-4975-03) and incubated at 37°C for 4 h before antibody staining. The following antibodies were used for mouse flow cytometry: Alexa Fluor® 700 anti-mouse CD45 [Clone: 30-F11] (BioLegend, cat: 103128), PE CF594 anti-mouse Cd3e [Clone: 145 2C11] (BD, cat: 562286), PE-Cyanine5 anti-mouse TCR gamma/delta [Clone: GL-3] (Invitrogen, cat: 15-5711-81), PE anti-mouse IL-17A [Clone: TC11-18H10.1] (BioLegend, cat: 506904).
Instrument	Samples were run on a BD Fortessa (BD Pharmingen).
Software	Data were analyzed using FlowJo software (Treestar, version 10.6.2).
Cell population abundance	After FACs, cells were directly collected and input for scRNA-seq, and the cell population has been verified by subsequent analysis (>95%).

Gating strategy	The total CD45+ live cell number was determined in the original cell suspension (SSC vs. FSC gating to exclude debris;FSC-H vs. FSC-A gating to exclude doublets; DAPI vs. FSC-A gating to exclude dead cells.) and used for total quantification of the cell number of each subset of interest defined by multi-parameter flow cytometry based on that subset's frequency relative to the CD45+ population.

☒ Tick this box to confirm that a figure exemplifying the gating strategy is provided in the Supplementary Information.
