# Supplementary material for: Tenascin C+ papillary fibroblasts facilitate neuro-immune interaction in a mouse model of psoriasis
Source: Nat Commun. 2023 Apr 10;14:2004. doi: 10.1038/s41467-023-37798-x (PMC10086024; doi:10.1038/s41467-023-37798-x)
Supplement: Supplementary file 3 — PeerReviewFile [file 41467_2023_37798_MOESM3_ESM.pdf]

nature portfolio

Peer Review File

Tenascin C+ Papillary Fibroblasts Facilitate Neuro-immune Interaction in Psoriasiform Skin Inflammation

Open Access This file is licensed under a Creative Commons Attribution 4.0 International License, which permits use, sharing, adaptation, distribution and reproduction in any medium or format, as long as you give appropriate credit to the original author(s) and the source, provide a link to the Creative Commons license, and indicate if changes were made. In the cases where the authors are anonymous, such as is the case for the reports of anonymous peer reviewers, author attribution should be to 'Anonymous Referee' followed by a clear attribution to the source work. The images or other third party material in this file are included in the article's Creative Commons license, unless indicated otherwise in a credit line to the material. If material is not included in the article's Creative Commons license and your intended use is not permitted by statutory regulation or exceeds the permitted use, you will need to obtain permission directly from the copyright holder. To view a copy of this license, visit http://creativecommons.org/licenses/by/4.0/.

REVIEWER COMMENTS</B>

Reviewer #1 (Remarks to the Author):

This manuscript by Cai et al found a cluster of TNC producing papillary fibroblasts which may promote neutrite outgrowth, promoting hyperinnervation and the development of psoriatic skin inflammation. Overall, the story is well written, easy to follow, and provide interesting data showing the involvement of TNC in nerve activation during psoriasis pathogenesis. However, there are several weaknesses of the study that the authors need to address. Please see specific comments below:
1. What is the mechanism by which neuron cells ae activated by TNC? What is the receptor that is expressed in the neuron to sense TNC? What are the downstream signaling event?
2. The authors showed that ablation of TNC not only inhibited axonogenesis, but also inhibited keratinocyte proliferation and T cell infiltration. So among these cell types (neuron, keratinocytes and T cells), what is the direct downstream target of TNC? Can blocking nerve activation prevent the inflammatory phenotype in TNC cKO mice?
3. Figure 6 investigated the interaction between neuron and delta gamma T cells, which seems to be quite disconnected from the rest of the study. How are delta gamma T cells altered in the TNC cKO mice? How can innervation alter these changes?
4. Why use Col1a2Cre instead of PDGFRACre to ablate TNC specifically in fibroblasts?
5. Figure 3d showing co-localization of betaIII tubulin+ nerves with fibroblasts should be performed using TNC lineage cells (in Tomado as shown in Fig. 3b-c) instead of Pdgfra lineage cells to support the conclusion that TNC promotes axonogenesis.
6. As shown in Figure 5H, betaIII tubulin+ nerves were primarily detected in the epidermis but not in the papillary dermis, where TNC was located. It is possible that TNC from papillary dFBs may act directly on epidermal keratinocytes, which in turn alter axonogenesis
7. It does not make sense for delta gamma T cells to be enriched with genes associated with postsynaptic pathways (Fig. 6F), such as postsynapse organization and vesicle mediated transport in synapse, which should be neuron specific. What are the specific genes? Please list these out and validate key representative gene expression by staining or other approach.

Reviewer #2 (Remarks to the Author):

In this manuscript Cai et al. report about a papillary fibroblast subset that arises upon psoriasiform skin inflammation and secretes tenascin-C (TNC), which promotes neurite outgrowth and innervation. The authors used an imiquimod-induced psoriasis mouse model and performed scRNA-seq. They identified several fibroblast subsets (13), one of them expressing Tnc and Coch, located specifically at the dermo-epithelial junction. GO pathway analysis revealed a possible function in neuromodulation of this fibroblast subset in inflammatory conditions. They also show TNC expression in human tissue. They performed in vitro co-culture assays with DRG neurons and TNC-overexpressing NIH-3T3 cells, which resulted in increased axonogenesis and denser neurite networks. They further knocked-out TNC in fibroblasts, which ameliorated the psoriasis phenotype upon imiquimod treatment (reduced skin thickness, reduced inflammation, reduced innervation). Moreover, scRNAseq was performed on immune cells of imiquimod-treated skin, the analysis of which suggested that gdTcells are the major source of IL17, and that the inflammation is regulated by neuronal signals. They further showed that IL17+ ydTcells increased in number upon imiquimod treatment and were in contact with cutaneous nerves in the early phase of

inflammation.
This study adds knowledge to the pathogenesis of psoriasis and highlights the role of fibroblasts and neurons in the inflammatory process. The methodology is sufficiently described, experiments seem to be well executed, and the conclusions are mostly supported by the data.
Major comments:
1. Are fibroblasts the only source of TNC? The lineage tracing with Tnc-DreER could also mark immune cells. This should be shown either by co-stainings or as a UMAP from the scRNA-Seq data.

2. Is TNC induced by IL17? To exclude that imiquimod directly induces TNC in fibroblasts, could the authors treat primary fibroblasts with the drug?
3. Fig. 5: what is the effect of TNC ablation on fibroblast themselves? Do they display reduced proliferation/ migration/ increased apoptosis?
Does genetic ablation of TNC in fibroblasts reduce axonogensis and neurite density in vitro? Does fibroblast-specific deletion of TNC effect skin morphology/function?
4. Fig. 1: What is the labelling efficiency upon tamoxifen treatment? In Figure 1g it looks as if the labelling is less than 50%. Since scRNA-Seq was performed on tomato+ cells, the data might not represent the full repertoire of cells.
Fig. 1d: why is no tomato signal detected on the vehicle-treated/untreated skin area
5. Fig1/Suppl. Fig. 1: the cluster "Myofibroblast" displays Acta1 expression but also Desmin expression. Could these cells also be pericytes or vSMCs oder Dermal sheath? A heatmap with more top-regulated genes for all clusters would be helpful.

Minor comment:
in Fig. 6b, not all clusters are labelled. Is cluster 8 comprised of gdTcells?

Reviewer #3 (Remarks to the Author):

Using scRNAseq, the authors discovered a fibroblast subset marked by tenascin-C (TNC) in an imiquimod-induced psoriasis mouse model. Fibroblast-specific TNC ablation suppressed hyperinnervation and reduced skin inflammation. In imiquimod-induced skin inflammation, confocal microscope revealed a close relationship between Nav1.8 neurons and IL17A T-cells. The authors claimed that TNC+ fibroblast subset is involved in neurite outgrowth via TNC and, as a result, facilitates the formation of neuro-immune synapses in psoriasis. The impact of TNC-expressing fibroblast cluster on psoriasis appears to be partial based on the skin thickness results, but the authors found a unique mechanism behind psoriasis. I have several major concerns.

Major
If TNC released from fibroblasts is involved in neurite outgrowth, neurite outgrowth in imiquimod-treated skin would be directed toward the fibroblasts. However, neurite outgrowth is directed toward the upper layer of the epidermis. TNC may act on immune cells and keratinocytes and promote skin inflammation. As a result, fibroblast-specific TNC ablation indirectly inhibits neurite outgrowth.
What is the receptor for TNC expressed by mouse sensory neurons? Three integrins were proposed by the authors, and three papers were cited (alpha 7, 8, and 9). Alpha 7 was confirmed only in CNS. Alpha 8 was only tested in chick motor and sensory neurons. Alpha 9 was not expressed in rat sensory neurons.
The title is overstated. Only the imiquimod-psoriasis model was tested by the authors. TNC-marked fibroblast clusters were reported in two papers (Theranostics 2020; 10(23): 10483-10497 and J Allergy Clin Immunol 2020;145:1615-28.). Please cite these papers and

discuss whether the clusters defined in this manuscript are the same.
TNC expression is significantly lower in mouse psoriasis skin than in human psoriasis skin (Fig. 3c). TNC localization differs in mice and is not limited to the dermal epidermal junction. The epidermal innervation of TNC-tdTomato mice appears to be reduced.
How did you draw the dermal epidermal junction?
Please explain in detail how you captured images of Nav1.8 neurons and IL17A T-cells. For example, how long was the z-axis step?

Minor
Line 94: Why CD45- cells were analyzed?
Line 122: Inaccurate description. Expression was not generally higher.
Line 127: Suppl Fig.2g is not immunofluorescent staining
Line 140: The image does not appear to be papillary dermis.
Line 199-200: Inaccurate description. You did not demonstrate that TNC expression levels are correlated with the severity of the inflammation.
Figs. 5J and 6h. Images are blurry.

Point-by-point Response

We thank the reviewers for their critical evaluation, constructive comments, and valuable suggestions of our manuscript entitled "Papillary Fibroblasts Expressing Tenascin C Define a Stromal Niche that Facilitates Neuro-immune Synapse Formation and Promotes Skin Inflammation" (NCOMMS-22-31227A-Z). Based on their comments, we have performed additional experiments and carefully revised our manuscript accordingly. The point-by-point responses to the reviewers' comments are provided in the following.

Reviewer #1 (Remarks to the Author):

This manuscript by Cai et al found a cluster of TNC producing papillary fibroblasts which may promote neutrite outgrowth, promoting hyperinnervation and the development of psoriatic skin inflammation. Overall, the story is well written, easy to follow, and provide interesting data showing the involvement of TNC in nerve activation during psoriasis pathogenesis. However, there are several weaknesses of the study that the authors need to address. Please see specific comments below:

1. What is the mechanism by which neuron cells are activated by TNC? What is the receptor that is expressed in the neuron to sense TNC? What are the downstream signaling events?

   Re: We thank the reviewer for these valuable questions and have included additional results to address concerns **(revised manuscript Line 175-188)**.

   (1) Several integrins have been identified as TNC receptors on neuronal membranes that mediate neurite outgrowth, including α7β1, α8β1, and α9 *J Neurosci* **29**, 5546-5557 (2009); *J Neurosci* **24**, 238-247 (2004); *Neuron* (1995). Based on a published database (GSE131230) *Neuron* **103**, 598-616 e597 (2019), we evaluated the expression of integrin subunits interact with TNC in dorsal root ganglion (DRG) neuron subtypes and found that α7β1 was dominantly expressed in DRG nociceptors **(Supplementary Fig. 4a)**. By qPCR analysis, we found that among all integrin subunits, α7β1 was significantly overexpressed by primary DRG neurons of imiquimod (IMQ)-induced mice compared with that of untreated (UT) mice **(Supplementary Fig. 4b)**, indicating they may participate in inflammation-induced neuropathy.

   (2) ERK signaling pathway plays an essential role in integrin-mediated neurite outgrowth *Cell Res* **22**, 954-972 (2012); *Development* **131**, 3433-3444 (2004); *Nature* **424**, 398-405 (2003). Our results showed that recombinant TNC (rTNC) strongly induced neurite outgrowth and ERK1/2 phosphorylation of primary DRG neurons in a dose-dependent manner **(Supplementary Fig. 4c, d)**. Besides, ERK agonist butylhydroquinone (TBHQ) further promoted TNC-induced neurite anomalous bifurcation, which was strongly suppressed by ERK inhibitor AZD6244 **(Supplementary Fig. 4e)**. These results suggested that the pro-axonogenesis

capacity of TNC is ERK-signaling dependent.

2. The authors showed that ablation of TNC not only inhibited axonogenesis, but also inhibited keratinocyte proliferation and T cell infiltration. So among these cell types (neuron, keratinocytes and T cells), what is the direct downstream target of TNC? Can blocking nerve activation prevent the inflammatory phenotype in TNC cKO mice?

Re: We followed the reviewer's constructive suggestions and examined the effect of denervation on skin inflammation phenotypes of IMQ-induced *Tnc*$^{fl/fl}$ (Floxed) and *Col1a2*^{CreER}*Tnc*$^{fl/fl}$ (cKO) mice **(revised manuscript Line 224-240)**.

(1) Denervation of peripheral nociceptors with resiniferatoxin (RTX) *Nature* **510**, 157-161 (2014) strongly blocked nociception **(Fig. 6a)**, and significantly suppressed epidermal hyperplasia in both IMQ-induced Floxed and cKO mice **(Fig. 6b-c)**. Notably, exfoliation was commonly seen after denervation (**Fig. 6b**), suggesting that peripheral neurons play essential roles in regulating epidermal integrity and function.

(2) Besides, fibroblast-conditional TNC knockout significantly decreased CD3e^{+} T cells especially γδT cell infiltration into skin lesions compared with Floxed mice. After denervation, the absolute number of γδT cells dramatically reduced, and are comparable between denervated cKO and Floxed mice **(Fig. 6d-f)**.

These results demonstrated that peripheral nociceptive sensory neurons served as a pivotal downstream of TNC and upstream of epidermal hyperplasia and immune cell infiltration during skin inflammation.

3. Figure 6 investigated the interaction between neuron and delta gamma T cells, which seems to be quite disconnected from the rest of the study. How are delta gamma T cells altered in the TNC cKO mice? How can innervation alter these changes?

Re: We appreciate the reviewer's valuable comments and have now included additional results **(revised manuscript Line 232-238)**.

(1) Dermal γδT cells are the major source of type 17 cytokines in IMQ-induced psoriasiform lesions. Fibroblast-conditional TNC knockout showed little effect on conventional T cells but significantly decreased dermal γδT cell infiltration in psoriasiform lesions compared with Floxed mice **(Fig. 6d-e)**. The IL-17A-producing capacity of γδT cells in TNC cKO mice slightly decreased but showed no significance in compared with Floxed mice **(Fig. 6f-g)**.

(2) The proportions of γδT cells dramatically reduced and are comparable between denervated cKO and Floxed mice **(Fig. 6d-e)**. Meanwhile, little effect of denervation on IL-17A-producing capacity of γδT cells was observed **(Fig. 6f-g)**.

Based on these results, the interactions between peripheral nerves and γδT cells during skin inflammation were further investigated.

4. Why use *Col1a2*^{Cre} instead of *Pdgfra*^{Cre} to ablate TNC specifically in fibroblasts?

Re: We thank the reviewer's careful consideration. As Dre does not recognize loxP locus, the *Pdgfra*^{DreER} mouse strain used for lineage tracing cannot be directly crossed with *Tnc*$^{fl/fl}$ mouse strain to generate conditional knock-out (cKO) mice. Thus, a previously well-established *Col1a2*^{CreER} strain was used instead for *Tnc*-specific ablation in fibroblasts. *Col1a2* has widespread expression and higher transcriptional activity among fibroblast-lineage cells (see below), making the *Col1a2*^{CreER} strain more suitable for the cKO strategy.

[REDACT]

5. Figure 3d showing co-localization of betaIII tubulin+ nerves with fibroblasts should be performed using TNC lineage cells (in Tomato as shown in Fig. 3b-c) instead of Pdgfra lineage cells to support the conclusion that TNC promotes axonogenesis.

Re: We followed the reviewer's advice and performed whole-mount staining of peripheral nerves with *Tnc*-tracing mice. Since TNC-tdTomato signal was barely detected in untreated mouse skin, a representative image of stained IMQ-induced lesional skin was presented **(Fig. 3e)**.

6. As shown in Figure 5H, betaIII tubulin+ nerves were primarily detected in the epidermis but not in the papillary dermis, where TNC was located. It is possible that TNC from papillary dFBs may act directly on epidermal keratinocytes, which in turn alter axonogenesis.

Re: We thank the reviewer for these valuable comments and have now included the additional data showing the skin innervation features and the importance of peripheral neurons for epidermal integrity and function.

(1) Figure 5H was captured with high power lens to focus epidermal innervation. As shown in the illustration (see below, left) *Nature* 445, 858-865 (2007) and the whole mount staining images of IMQ-induced mouse skin (see below, right), βIII tubulin^{+} nerve fibers are widespread in the skin and are stretching from the dermis into the epidermis.

[REDACT]

(2) After denervation of peripheral nociceptors with resiniferatoxin (RTX) *Nature* **510**, 157-161 (2014), the IMQ-induced epidermal hyperplasia, inflammatory cell infiltration, and skin inflammation were greatly restrained **(Fig. 6b-e)**, suggesting peripheral sensory nerves play central roles in promoting epidermal reactive acanthosis and cutaneous inflammatory responses. Although we cannot rule out the effect of TNC on keratinocyte proliferation and differentiation, given the unique location of *Tnc*⁺ dermal fibroblast subset at the dermal-epidermal junction and its capacity for pro-axonogenesis, the peripheral sensory nerves serve as essential downstream of TNC and upstream of epidermal hyperplasia.

7. It does not make sense for delta gamma T cells to be enriched with genes associated with postsynaptic pathways (Fig. 6F), such as postsynapse organization and vesicle mediated transport in synapse, which should be neuron specific. What are the specific genes? Please list these out and validate key representative gene expression by staining or other approach.

Re: We indeed appreciate the reviewer for the detailed advice and have included additional results to address the reviewer's concerns.

(1) Vesicle-mediated transport in synapse happens not only in neurons but also in immune cells, and is a key process during immunological synapse formation *Front Immunol* 9, 684 (2018), such as antigen presentation, lytic granule secretion and *etc.* It was reported that trans-synaptic vesicles could be secreted by T cells to facilitate intercellular communications *Nat Commun* 13, 3460 (2022). Sustained interaction and synapse formation between Th17 cells and nerve fibers have also been published *Cell* **186**, 607-620 e617 (2023); *Immunity* **33**, 424-436 (2010). These studies support that T cells have intrinsic synapse formation and vesicle-mediated transport mechanisms and have interactions with neurites.

(2) To address the reviewer's concern, we reorganized GSEA results. The complete enriched gene/pathway lists were attached in **Source Data related to Fig. 7f** (Il17a pos T versus Il17a neg T_GSEA analysis.xlsx) and original GSEA output files were deposited at https://data.mendeley.com/datasets/jgmkgbh875/1 for your reference. Each line represents expression metadata of a single γδT cell in the heatmap below. Although expression level varies among cells, *Il17a*⁺ γδT cells showed generally higher expression levels of core enrichment genes compared with *Il17a*⁻ γδT cells.

[REDACT]

(3) Two representative genes that enriched in differentially expressed pathways of *Il17a*$^+$ versus *Il17a*$^-$ γδT cells, *Flna* and *Arf6*, are discussed below.

a) *Flna* (enriched Regulation_of_neuron_projection_regeneration pathway) encodes an actin-binding protein filamin A (FLNA) that is involved in cytoskeleton remodeling. FLNA participates in neuronal migration, and its overexpression in neurons leads to abnormal dendritic patterning *Neuron* **52**, 789-801 (2006); *Neuron* **84**, 78-91 (2014). In T cells, FLNA is required for T cell activation and localized at T cell-antigen presenting cell immune-synapses (see below, left) *J Immunol* 177, 1721-1728 (2006).

b) *Arf6* (enriched in Vesicle_mediated_transport_in_synapse and Postsynapse_organization pathways) encodes a small guanine nucleotide-binding protein that is localized to the plasma membrane and regulates vesicular trafficking. *Arf6* involves in neuron regeneration and migration *Small GTPases* **11**, 392-401 (2018); *Proc Natl Acad Sci U S A* **111**, 2337-2342 (2014). During T cell-antigen presenting cell immunologic synapse formation, *Arf6* is required for efficient conjugation via regulating internalization and recycling of clathrin-independent endocytosis cargo proteins (see below, right) *J Cell Sci* 130, 2405-2415 (2017).

[REDACT]

These studies revealed that T cells and neurons share similar factors and pathways in synapse organization. Due to the dynamic and transient feature of synapse formation, whether these factors participate in the neuroimmune synapse formation between *Il17a*$^{+}$ T cells and peripheral sensory neurons during skin inflammation, and more importantly, to what extent these key factors may influence the disease severity, remain to be further elucidated and beyond what we focus in this research.

Reviewer #2 (Remarks to the Author):

In this manuscript Cai et al. report about a papillary fibroblast subset that arises upon psoriasiform skin inflammation and secretes tenascin-C (TNC), which promotes neurite outgrowth and innervation. The authors used an imiquimod-induced psoriasis mouse model and performed scRNA-seq. They identified several fibroblast subsets (13), one of them expressing Tnc and Coch, located specifically at the dermo-epithelial junction. GO pathway analysis revealed a possible function in neuromodulation of this fibroblast subset in inflammatory conditions. They also show TNC expression in human tissue. They performed in vitro co-culture assays with DRG neurons and TNC-overexpressing NIH-3T3 cells, which resulted in increased axonogenesis and denser neurite networks. They further knocked-out TNC in fibroblasts, which ameliorated the psoriasis phenotype upon imiquimod treatment (reduced skin thickness, reduced inflammation, reduced innervation). Moreover, scRNAseq was performed on immune cells of imiquimod-treated skin, the analysis of which suggested that gdTcells are the major source of IL17, and that the inflammation is regulated by neuronal signals. They further showed that IL17+ ydTcells increased in number upon imiquimod treatment and were in contact with cutaneous nerves in the early phase of inflammation.

This study adds knowledge to the pathogenesis of psoriasis and highlights the role of fibroblasts and neurons in the inflammatory process. The methodology is sufficiently described, experiments seem to be well executed, and the conclusions are mostly supported by the data.

Major comments:

1. Are fibroblasts the only source of TNC? The lineage tracing with Tnc-DreER could also mark immune cells. This should be shown either by co-stainings or as a UMAP from the scRNA-Seq data.

   Re: We thank the reviewer for these valuable suggestions and examined the expression of *Tnc* in keratinocytes and immune cells.

   (1) We integrated our single-cell RNA-seq data of keratinocytes/CD45 (*Ptprc*)$^+$ immune cells/*Pdgfra*-lineage stromal cells from untreated and imiquimod-induced mouse skin. As shown in the UMAP/Violin plots below, a subset of dermal fibroblasts (Fb_5, marked by red dotted line) is the major source of TNC in mouse psoriasiform lesions, and keratinocytes and immune cells showed little expression of *Tnc* with/without IMQ stimulation.

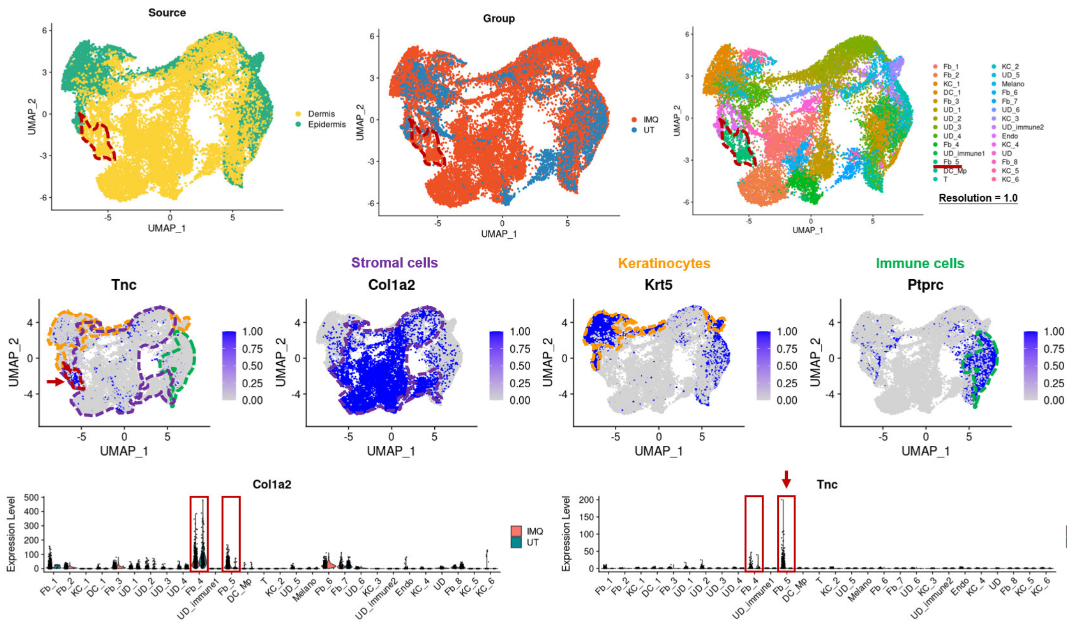

(2) Besides, from our whole-mount fluorescent images of *Tnc*^{DreER}-tdTomato mice, tdTomato did not label keratinocytes in untreated or IMQ-induced mouse skin **(Fig. 3d)**, and the morphology and localization of tdTomato^{+} cells are more likely fibroblasts in the hair follicle and at epidermal-dermal junction in the papillary dermis.

2. Is TNC induced by IL17? To exclude that imiquimod directly induces TNC in fibroblasts, could the authors treat primary fibroblasts with the drug?

Re: We thank for the reviewer's detailed suggestion and have performed additional experiments to address this concern. To evaluate whether TNC could be induced by imiquimod or IL-17, we add imiquimod (IMQ, 2μg/mL), resiquimod (R848, a commonly used TLR7/8 agonist, 1μg/mL), rhIL-17A (10ng/mL), as well as several other inflammatory cytokines (10ng/mL) which have been reported as TNC inducers into primary mouse dermal fibroblast culture medium. Cell lysates were collected after 24-hour culture and Western blotting showed that TNC can be induced by various inflammatory cytokines including IL-17A, while IMQ and R848 do not directly induce TNC expression **(Source Data Fig.1a)**.

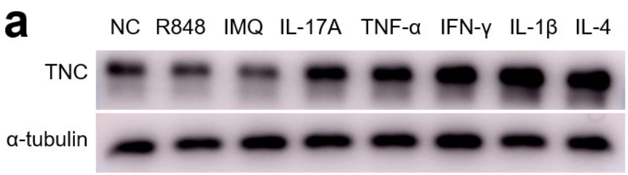

3. Fig. 5: what is the effect of TNC ablation on fibroblast themselves? Do they display reduced proliferation/ migration/ increased apoptosis?
Does genetic ablation of TNC in fibroblasts reduce axonogenesis and neurite density *in vitro*?
Does fibroblast-specific deletion of TNC effect skin morphology/function?

Re: We thank the reviewer for these valuable comments and have now included additional data to address concerns.

(1) Mouse primary fibroblasts ablated TNC displayed reduced proliferation/migration **(Source Data Fig.1b, e)**, increased early apoptosis **(Source Data Fig.1c, d)**, and showed impaired pro-axonogenesis capacity **(Source Data Fig.1f**, fibroblasts are labeled by white arrowheads**)**.

(2) Besides, fibroblast-specific deletion of TNC do not affect skin morphology and function at homeostatic states, as shown by histology analysis **(Fig. 6b, c)** and daily trans-epidermal water loss (TEWL) score measurement **(Source Data Fig.1g)** of shaved *Tnc*$^{fl/fl}$ or *Col1a2*^{CreER}*Tnc*$^{fl/fl}$ mouse skin two weeks after tamoxifen-induced Cre-mediated recombination.

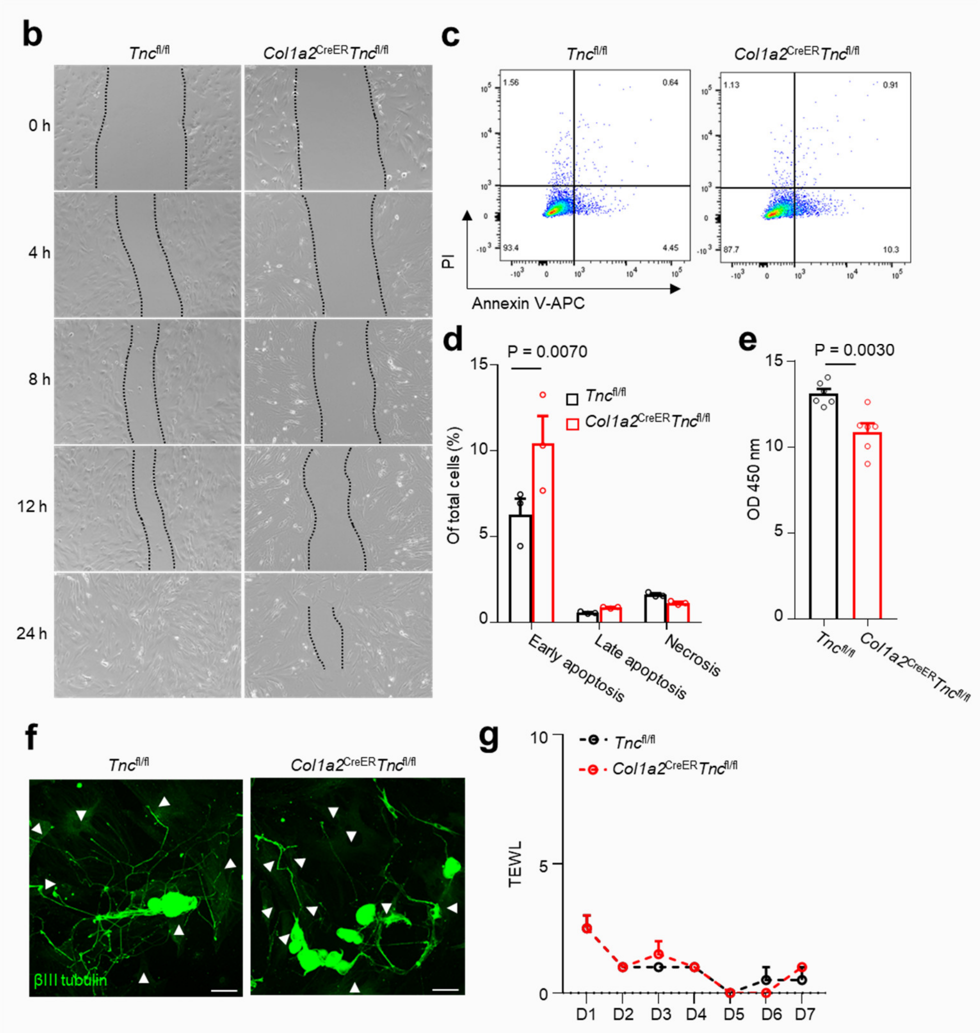

4. Fig. 1: What is the labelling efficiency upon tamoxifen treatment? In Figure 1g it looks as if the labelling is less than 50%. Since scRNA-Seq was performed on tomato+ cells, the data might not represent the full repertoire of cells.

Re: We thank the reviewer for the careful consideration and have now included additional results **(Fig. 1b, c, revised manuscript Line 83-84)**.

After 5-day tamoxifen injection and 2-week wash out, single-cell suspensions were generated from UT/IMQ-treated Pdgfra^{DreER}-tdTomato mouse skin by enzyme digestion and subjected to fluorescence-activated cell sorting (FACS) to exclude doublets, debris, and DAPI^{+} dead cells. TdTomato labeled over 75% fibroblasts in untreated mouse skin upon tamoxifen treatment, suggesting good efficiency in fibroblast labeling. In IMQ-induced mouse skin, the labeling percentage fell down to about 40%, this may due to inflammation-induced proliferation and differentiation of fibroblasts. Given that tdTomato genetically labeled fibroblast during disease course, we think our scRNA-seq data represented the majority features of fibroblasts.

Fig. 1d: why is no tomato signal detected on the vehicle-treated/untreated skin area?

Re: We thank the reviewer for the careful consideration. For reporter mice imaging, tdTomato signal can also be detected on the untreated/vehicle-treated skin area at a lower level. Fig. 1d was adjusted from the original image below at a different exposure time, and the fluorescence activity of ROIs was calculated and quantified. Detailed values were provided in **Source Data related to Fig.1e**.

Schematic diagram

[REDACT]

Original captured image | **Modified image in Figure.1d**

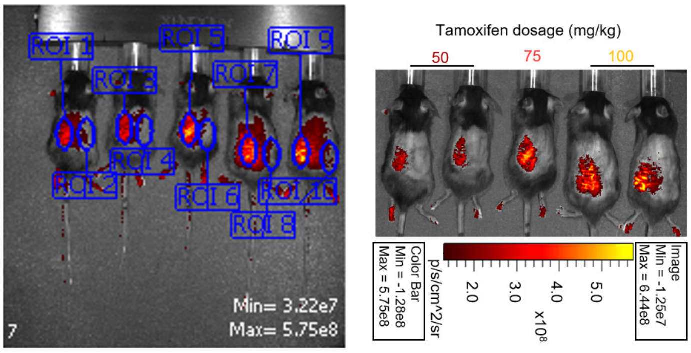

5. Fig1/Suppl. Fig. 1: the cluster “Myofibroblast” displays Acta1 expression but also Desmin expression. Could these cells also be pericytes or vSMCs or Dermal sheath? A heatmap with more top-regulated genes for all clusters would be helpful.

   Re: We followed the reviewer’s valuable suggestion and have included the results.

   (1) A heatmap of top-ten cluster marker genes is present below, and a table sheet containing all cluster-specific marker genes was attached to the **Source Data related to Supplementary Fig.1e**.

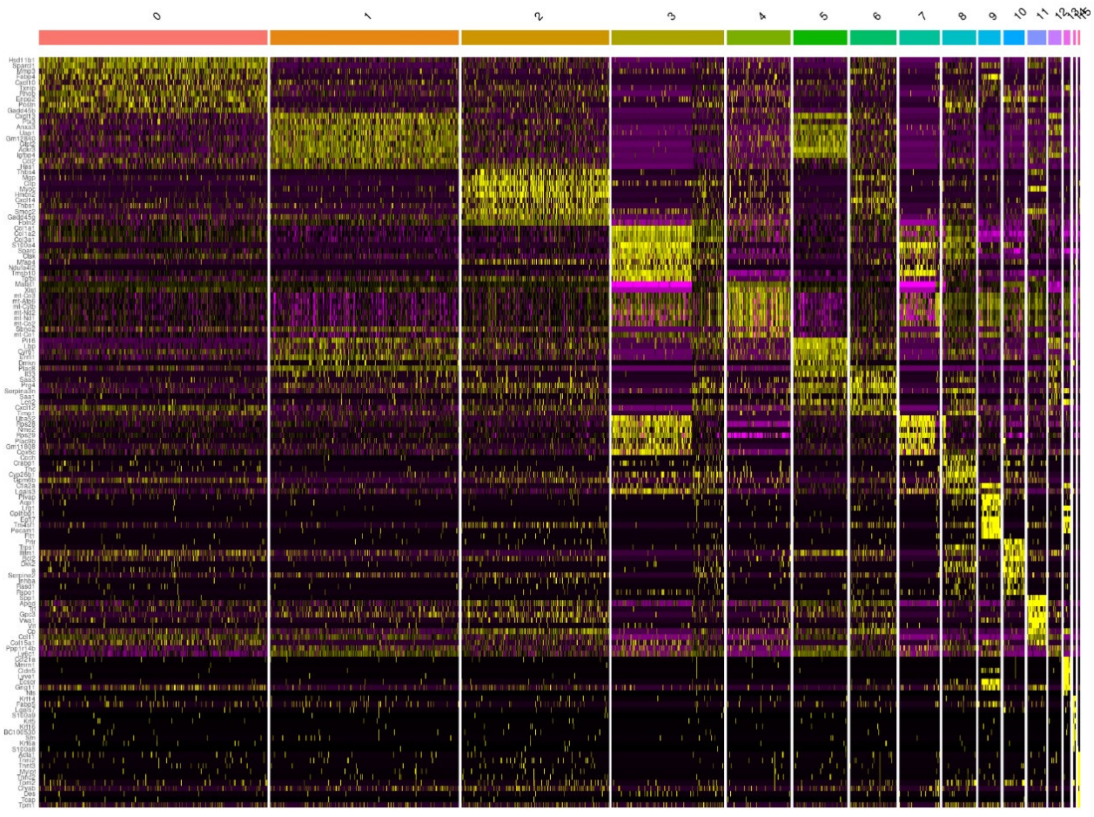

(2) As shown in the small heatmap below, the cluster "Myofibroblast" neither expressed *Pdgfrb* for pericytes *Am J Physiol Lung Cell Mol Physiol* 315, L991-L1002 (2018), Cnn2 for SMCs *Gene* 585, 143-153 (2016), nor *Col11a1*/*Myl9*/*Tnmd* for Dermal sheath populations *Front Genet* 12, 797747 (2021), but specifically expressed genes associated with myofibroblasts, including: *Tcap*/*Myl1*/*Myoz1*, *etc*.

[REDACT]

Minor comment:

In Fig. 6b, not all clusters are labelled. Is cluster 8 comprised of gd T cells?

Re: We thank the reviewer for the careful consideration. As shown in **Supplementary Fig. 5b**, cells in cluster 7 showed no identical gene signature, makes it difficult to be defined and was not labelled in **Fig. 6b**. Cluster 2/8 highly expressed Cd3e/Cd3g, showed a mixed gene signature of conventional T/ NK T (Cd7/Nkg7)/γδT (Trdc) and were combinedly labeled as T cells. Cluster 8, marked by *Trdc*, is mainly comprised of γδT cells and is the major source of pathogenic type 17 cytokines **(Fig. 6b-c)**.

Reviewer #3 (Remarks to the Author):

Using scRNAseq, the authors discovered a fibroblast subset marked by tenascin-C (TNC) in an imiquimod-induced psoriasis mouse model. Fibroblast-specific TNC ablation suppressed hyperinnervation and reduced skin inflammation. In imiquimod-induced skin inflammation, confocal microscope revealed a close relationship between Nav1.8 neurons and IL17A T-cells. The authors claimed that TNC+ fibroblast subset is involved in neurite outgrowth via TNC and, as a result, facilitates the formation of neuro-immune synapses in psoriasis. The impact of TNC-expressing fibroblast cluster on psoriasis appears to be partial based on the skin thickness results, but the authors found a unique mechanism behind psoriasis. I have several major concerns.

Major

1. If TNC released from fibroblasts is involved in neurite outgrowth, neurite outgrowth in imiquimod-treated skin would be directed toward the fibroblasts. However, neurite outgrowth is directed toward the upper layer of the epidermis. TNC may act on immune cells and keratinocytes and promote skin inflammation. As a result, fibroblast-specific TNC ablation indirectly inhibits neurite outgrowth.

   Re: We thank the reviewer for these valuable comments and have now included the additional data showing the skin innervation features and the importance of peripheral neurons for epidermal integrity, function and inflammatory immune response.

   (1) As shown in the illustration (see below, left) *Nature* 445, 858-865 (2007) and the whole mount staining images of IMQ-induced mouse skin (see below, right), peripheral nerve fibers are stretching from the dermis into the epidermis. The orientation of peripheral nerve towards epidermis may due to their function in environmental perception and modulation of epidermis and its appendages. Epidermal cells may also secrete neuro-regulation factors and promote neurite outgrowth.

[REDACT]

   (2) Our *in vitro* experiments involving only TNC-expressing fibroblasts **(Fig. 4d-i)** or recombinant TNC **(Supplementary Fig. 4c)** and DRG neurons have verified the direct pro-axonogenesis effect of TNC. Besides, we co-cultured DRG neurons with primary dermal fibroblasts from *Tnc*$^{fl/fl}$ (Floxed) or *Col1a2*^{CreER}*Tnc*$^{fl/fl}$ (cKO) mice, and found dermal fibroblasts ablated TNC displayed impaired pro-axonogenesis capacity **(**see below, **Source Data Fig.1f**, fibroblasts are labeled by white

arrowheads).

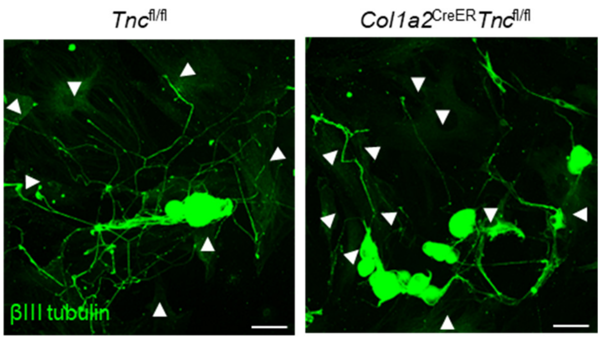

(3) Fibroblast-specific TNC ablation significantly suppressed innervation of inflamed epidermis *in vivo* **(Fig. 5h, j)**. Denervation of peripheral nociceptors with resiniferatoxin (RTX) *Nature* **510**, 157-161 (2014) greatly restrained IMQ-induced epidermal hyperplasia, inflammatory cell infiltration and skin inflammation in both Floxed and cKO mice **(Fig. 6b-e)**. These results suggested that TNC exacerbates skin inflammation through peripheral neurons. Although we cannot rule out the effect of TNC on keratinocyte proliferation and immune cell migration, peripheral sensory nerves serve as essential downstream of TNC.

2. What is the receptor for TNC expressed by mouse sensory neurons? Three integrins were proposed by the authors, and three papers were cited (alpha 7, 8, and 9). Alpha 7 was confirmed only in CNS. Alpha 8 was only tested in chick motor and sensory neurons. Alpha 9 was not expressed in rat sensory neurons.

Re: We thank the reviewer for these important comments and have included additional results to address concerns **(Supplementary Fig. 4a, b, revised manuscript Line 171-177)**.

Based on a published database (GSE131230) *Neuron* **103**, 598-616 e597 (2019), we evaluated the expression of integrin subunits that interact with TNC in dorsal root ganglion (DRG) neuron subtypes and found that α7β1 was dominantly expressed in nociceptors **(Supplementary Fig. 4a)**. By qPCR analysis, we found that among all integrin subunits, α7β1 significantly overexpressed by DRG neurons of IMQ-induced mice compared with that of UT mice **(Supplementary Fig. 4b)**. These results suggested that integrin α7β1 may participate in inflammation-induced neuropathy of IMQ-induced lesions.

3. The title is overstated. Only the imiquimod-psoriasis model was tested by the authors. TNC-marked fibroblast clusters were reported in two papers (Theranostics 2020; 10(23): 10483-10497 and J Allergy Clin Immunol 2020;145:1615-28.). Please cite these papers and discuss whether the clusters defined in this manuscript are the same.

Re: We followed the reviewer's valuable comments and have cited and discussed these two studies **(revised manuscript Line 279-281)**.

(1) We analyzed the *Tnc*$^{+}$ fibroblast cluster defined in our manuscript and the

expression level of other marker genes from published scRNA-seq databases of an immuno-dysregulated psoriatic mouse model *Theranostics* **10**, 10483-10497 (2020) and of human atopic dermatitis lesions J Allergy Clin Immunol 145, 1615-1628 (2020). We found that *Tnc* itself defined a more specific pro-inflammatory fibroblast subset which shares similar gene profiles with previous studies (see violin plots below). Meanwhile, *Tnc*$^+$ fibroblasts co-expressed *Ccl2* and *Ccl19*, suggesting their chemotactic effects on immune cells.

[REDACT]

(2) Besides imiquimod (IMQ)-induced psoriasis mouse model, analysis of scRNA-seq database (GSE150672, **Supplementary Fig. 2d-f**), spatial transcriptomics **(Fig. 1i)**, and immunofluorescent staining images **(Fig. 1j)** of normal/ psoriatic human skin were also included in our study, and have demonstrated a similar transcriptional feature, expression pattern and location of TNC to what we found in IMQ mouse model. Our preliminary data showed that TNC can be induced at epidermal-dermal junction in multiple inflammatory skin diseases (see below), including psoriasis (PsO), vitiligo (Viti) and alopecia areata (AA). Our study emphasized the pathogenic role of *Tnc*$^+$ papillary fibroblasts in promoting neurite outgrowth and skin inflammation, and can be applicated for the treatment of psoriasis and other inflammatory skin diseases.

[REDACT]

4. TNC expression is significantly lower in mouse psoriasis skin than in human psoriasis skin (Fig. 3c). TNC localization differs in mice and is not limited to the dermal epidermal junction. The epidermal innervation of TNC-tdTomato mice appears to be reduced.

Re: We appreciate the reviewer's careful evaluation.

(1) Immunofluorescent staining showed that mouse psoriasiform lesion exhibited similar TNC expression pattern to that of human psoriatic lesion at epidermal-dermal junction (EDJ), but the staining intensity was relatively lower **(Fig. 2g, j)**, this may due to the intrinsic structure and differences between mouse and human skin.

(2) Original **Fig. 3c** has been replaced by whole-mount images of *Tnc*^{DreER}-tdTomato mouse skin to display *Tnc*$^{+}$ fibroblast distribution and innervation pattern **(Fig. 3d, e)**. Fluorescent images of *Tnc*^{DreER}-tdTomato reporter mice showed that *Tnc*$^{+}$ fibroblasts localized at dermal papillae (DP) at homeostatic state, and were induced at EDJ during skin inflammation. It is worth noting that TNC protein can barely be stained at DP by immunofluorescent assay **(Fig. 2g)**, this discrepancy may due to a higher transcriptional sensitivity of reporter mice.

5. How did you draw the dermal epidermal junction?

Re: As can be seen from the H&E and immunofluorescent images below, the epidermis can be distinguished from the dermis by basal membrane where the cell nuclei are densely arranged.

[REDACT]

6. Please explain in detail how you captured images of Nav1.8 neurons and IL17A T-cells. For example, how long was the z-axis step?

   Re: For Nav1.8^{tdTomato}IL17^{GFP} dual reporter mice imaging, images were captured by Leica SP8 microscopy with HC PL APO CS2 40x/1.30 OIL objective, with a scan speed of 200 Hz and a step size of 1.0 μm. The X/Y acquiring format was set as 1024*1024, and Z-stack size was determined by specimen thickness respectively. These parameters have been added to the methods section **(revised manuscript Line 675-678)**.

Minor

Line 94: Why CD45- cells were analyzed?

Re: We appreciate the reviewer's careful consideration. The CD45^{-}tdTomato^{+} sorting strategy was used to purify fibroblast-lineage cells and rule out RFP or fibroblast debris uptake by phagocytic cells. Typically, fibroblasts do not express the myeloid gene CD45(*Ptprc*). As shown in the figure below, 99.3% of *Pdgfra*-tdTomato lineage cells are negative for CD45 staining.

[REDACT]

Line 122: Inaccurate description. Expression was not generally higher.

Re: We followed the reviewer's suggestion and refined our expression. "*TNC* showed upregulated expression level in human psoriatic skin fibroblasts compared with normal fibroblasts **(revised manuscript, Line 127-128)**."

Line 127: Suppl Fig.2g is not immunofluorescent staining

Re: We apologized for the inaccurate description. **Supplementary Fig. 2g** is a representative H&E staining image of the psoriatic skin section with both lesional and non-lesional areas, and the immunofluorescent staining images were shown in **Fig. 2j**. Related description in the manuscript has been modified **(revised manuscript, Line 131-133)**. "In human psoriatic skin with both lesional and non-lesional regions (Supplementary Fig. 2g), TNC showed escalating staining intensity from non-lesional to lesional area (Fig. 2j)."

Line 140: The image does not appear to be papillary dermis.

Re: We apologized for blurry images and have replaced this figure with whole-mount images of *Tnc*^{DreER}-tdTomato mouse skin with more distinct papillary dermis features **(Fig. 2d-e)**.

Line 199-200: Inaccurate description. You did not demonstrate that TNC expression levels are correlated with the severity of the inflammation.

Re: We followed the reviewer's suggestion and added a correlation analysis between TNC expression and skin acanthosis which reflect the disease severity in **(Supplementary Fig. 4f, revised manuscript Line 218-219)**.

Figs. 5J and 6h. Images are blurry.

Re: We apologized for blurry images and have replaced these figures by re-captured images of relevant specimens.

References

M. R. Andrews, S. Czvitkovich, E. Dassie, C. F. Vogelaar, A. Faissner, B. Blits, F. H. Gage, C. ffrench-Constant and J. W. Fawcett Alpha9 integrin promotes neurite outgrowth on tenascin-C and enhances sensory axon regeneration. *J Neurosci* **29**, 5546-5557 (2009).

M. L. Mercado, A. Nur-e-Kamal, H. Y. Liu, S. R. Gross, R. Movahed and S. Meiners Neurite outgrowth by the alternatively spliced region of human tenascin-C is mediated by neuronal alpha7beta1 integrin. *J Neurosci* **24**, 238-247 (2004).

K. V. Barbara Varnum-Finney, t, R. K. Ulrich Muller, Carey Backus, and a. L. F. R. Matthias Chiquet The Integrin Receptor α8β1 Mediates Interactions of Embryonic Chick Motor and Sensory Neurons with Tenascin-C. *Neuron* (1995).

Y. Zheng, P. Liu, L. Bai, J. S. Trimmer, B. P. Bean and D. D. Ginty Deep Sequencing of Somatosensory Neurons Reveals Molecular Determinants of Intrinsic Physiological Properties. *Neuron* **103**, 598-616 e597 (2019).

W. L. Lei, S. G. Xing, C. Y. Deng, X. C. Ju, X. Y. Jiang and Z. G. Luo Laminin/beta1 integrin signal triggers axon formation by promoting microtubule assembly and stabilization. *Cell Res* **22**, 954-972 (2012).

L. S. Campos, D. P. Leone, J. B. Relvas, C. Brakebusch, R. Fassler, U. Suter and C. ffrench-Constant Beta1 integrins activate a MAPK signalling pathway in neural stem cells that contributes to their maintenance. *Development* **131**, 3433-3444 (2004).

R. J. Pasterkamp, J. J. Peschon, M. K. Spriggs and A. L. Kolodkin Semaphorin 7A promotes axon outgrowth through integrins and MAPKs. *Nature* **424**, 398-405 (2003).

L. Riol-Blanco, J. Ordovas-Montanes, M. Perro, E. Naval, A. Thiriot, D. Alvarez, S. Paust, J. N. Wood and U. H. von Andrian Nociceptive sensory neurons drive interleukin-23-mediated psoriasiform skin inflammation. *Nature* **510**, 157-161 (2014).

E. A. Lumpkin and M. J. Caterina Mechanisms of sensory transduction in the skin. *Nature* **445**, 858-865 (2007).

V. Calvo and M. Izquierdo Imaging Polarized Secretory Traffic at the Immune Synapse in Living T Lymphocytes. *Front Immunol* **9**, 684 (2018).

P. F. Cespedes, A. Jainarayanan, L. Fernandez-Messina, S. Valvo, D. G. Saliba, E. Kurz, A. Kvalvaag, L. Chen, C. Ganskow, H. Colin-York, M. Fritzsche, Y. Peng, T. Dong, E. Johnson, J. A. Siller-Farfan, O. Dushek, E. Sezgin, B. Peacock, A. Law, D. Aubert, S. Engledow, M. Attar, S. Hester, R. Fischer, F. Sanchez-Madrid and M. L. Dustin T-cell trans-synaptic vesicles are distinct and carry greater effector content than constitutive extracellular vesicles. *Nat Commun* **13**, 3460 (2022).

M. Enamorado, W. Kulalert, S. J. Han, I. Rao, J. Delaleu, V. M. Link, D. Yong, M. Smelkinson, L. Gil, S. Nakajima, J. L. Linehan, N. Bouladoux, J. Wlaschin, J. Kabat, O. Kamenyeva, L. Deng, I. Gribonika, A. T. Chesler, I. M. Chiu, C. E. Le Pichon and Y. Belkaid Immunity to the microbiota promotes sensory neuron regeneration. *Cell* **186**, 607-620 e617 (2023).

V. Siffrin, H. Radbruch, R. Glumm, R. Niesner, M. Paterka, J. Herz, T. Leuenberger, S. M. Lehmann, S. Luenstedt, J. L. Rinnenthal, G. Laube, H. Luche, S. Lehnardt, H. J. Fehling, O. Griesbeck and F. Zipp In vivo imaging of partially reversible th17 cell-induced neuronal dysfunction in the course of encephalomyelitis. *Immunity* **33**, 424-436 (2010).

M. R. Sarkisian, C. M. Bartley, H. Chi, F. Nakamura, K. Hashimoto-Torii, M. Torii, R. A. Flavell and P. Rakic MEKK4 signaling regulates filamin expression and neuronal migration. *Neuron* **52**, 789-801 (2006).

L. Zhang, C. M. Bartley, X. Gong, L. S. Hsieh, T. V. Lin, D. M. Feliciano and A. Bordey MEK-ERK1/2-dependent FLNA overexpression promotes abnormal dendritic patterning in tuberous sclerosis independent of mTOR. *Neuron* **84**, 78-91 (2014).

K. Hayashi and A. Altman Filamin A is required for T cell activation mediated by protein kinase C-theta. *J Immunol* **177**, 1721-1728 (2006).

D. L. Johnson, J. Wayt, J. M. Wilson and J. G. Donaldson Arf6 and Rab22 mediate T cell conjugate formation by regulating clathrin-independent endosomal membrane trafficking. *J Cell Sci* **130**, 2405-2415 (2017).

B. Nieuwenhuis and R. Eva ARF6 and Rab11 as intrinsic regulators of axon regeneration. *Small GTPases* **11**, 392-401 (2018).

A. Falace, E. Buhler, M. Fadda, F. Watrin, P. Lippiello, E. Pallesi-Pocachard, P. Baldelli, F. Benfenati, F. Zara, A. Represa, A. Fassio and C. Cardoso TBC1D24 regulates neuronal migration and maturation through modulation of the ARF6-dependent pathway. *Proc Natl Acad Sci U S A* **111**, 2337-2342 (2014).

C. L. Wilson, S. E. Stephenson, J. P. Higuero, C. Feghali-Bostwick, C. F. Hung and L. M. Schnapp Characterization of human PDGFR-beta-positive pericytes from IPF and non-IPF lungs. *Am J Physiol Lung Cell Mol Physiol* **315**, L991-L1002 (2018).

R. Liu and J. P. Jin Calponin isoforms CNN1, CNN2 and CNN3: Regulators for actin cytoskeleton functions in smooth muscle and non-muscle cells. *Gene* **585**, 143-153 (2016).

J. M. D. Ahlers, C. Falckenhayn, N. Holzscheck, L. Sole-Boldo, S. Schutz, H. Wenck, M. Winnefeld, F. Lyko, E. Gronniger and A. Siracusa Single-Cell RNA Profiling of Human Skin Reveals Age-Related Loss of Dermal Sheath Cells and Their Contribution to a Juvenile Phenotype. *Front Genet* **12**, 797747 (2021).

C. Qie, J. Jiang, W. Liu, X. Hu, W. Chen, X. Xie and J. Liu Single-cell RNA-Seq reveals the transcriptional landscape and heterogeneity of skin macrophages in Vsir(-/-) murine psoriasis. *Theranostics* **10**, 10483-10497 (2020).

H. He, H. Suryawanshi, P. Morozov, J. Gay-Mimbrera, E. Del Duca, H. J. Kim, N. Kameyama, Y. Estrada, E. Der, J. G. Krueger, J. Ruano, T. Tuschl and E. Guttman-Yassky Single-cell transcriptome analysis of human skin identifies novel fibroblast subpopulation and enrichment of immune subsets in atopic dermatitis. *J Allergy Clin Immunol* **145**, 1615-1628 (2020).

REVIEWERS' COMMENTS

Reviewer #1 (Remarks to the Author):

The authors have adequately addressed my questions. I have no more questions.

Reviewer #2 (Remarks to the Author):

The authors have addressed all my concerns and added a tremendous amount of additional data in response to the comments of the other reviewers. The revised manuscript has improved substantially.

Reviewer #3 (Remarks to the Author):

By addressing the reviewers' concerns, the manuscript has been substantially improved.

Point-by-point Response

Reviewer #1 (Remarks to the Author):

The authors have adequately addressed my questions. I have no more questions.

Re: We thank the reviewer for the constructive suggestions which helped improve our research substantially.

Reviewer #2 (Remarks to the Author):

The authors have addressed all my concerns and added a tremendous amount of additional data in response to the comments of the other reviewers. The revised manuscript has improved substantially.

Re: We thank the reviewer for the careful evaluation and detailed advice which help us to improve the integrity of our research.

Reviewer #3 (Remarks to the Author):

By addressing the reviewers' concerns, the manuscript has been substantially improved.

Re: We appreciate the reviewer's careful evaluation and valuable comments which greatly helped improve our research quality.
